# Targeting Nuclear LSD1 to Reprogram Cancer Cells and Reinvigorate Exhausted T Cells via a Novel LSD1-EOMES Switch

**DOI:** 10.3389/fimmu.2020.01228

**Published:** 2020-06-16

**Authors:** Wen Juan Tu, Robert D. McCuaig, Abel H. Y. Tan, Kristine Hardy, Nabila Seddiki, Sayed Ali, Jane E. Dahlstrom, Elaine G. Bean, Jenny Dunn, Jade Forwood, Sofia Tsimbalyuk, Kate Smith, Desmond Yip, Laeeq Malik, Thiru Prasanna, Peter Milburn, Sudha Rao

**Affiliations:** ^1^Gene Regulation and Translational Medicine Laboratory, QIMR Berghofer Medical Research Institute, Brisbane, QLD, Australia; ^2^Melanie Swan Memorial Translational Centre, Faculty of Science and Technology, University of Canberra, Bruce, ACT, Australia; ^3^Inserm, U955, Equipe 16, Créteil, France; ^4^Université Paris Est, Faculté de Médecine, Créteil, France; ^5^Vaccine Research Institute (VRI), Créteil, France; ^6^Medical Oncology, St John of God Midland Public and Private Hospitals, Midland, WA, Australia; ^7^Anatomical Pathology, ACT Pathology, The Canberra Hospital, Canberra Health Services, Garran, ACT, Australia; ^8^ANU Medical School, College of Health and Medicine, The Australian National University, Canberra, ACT, Australia; ^9^The John Curtin School of Medical Research, The Australian National University, Canberra, ACT, Australia; ^10^School of Biomedical Sciences, Charles Sturt University, Wagga Wagga, NSW, Australia; ^11^Australian Synchtrotron - ANSTO, Clayton, VIC, Australia; ^12^Department of Medical Oncology, The Canberra Hospital, Canberra Health Services, Garran, ACT, Australia

**Keywords:** cancer, circulating tumor cells, EOMES, immunotherapy resistance, LSD1, PD-1, T cell exhaustion

## Abstract

Lysine specific demethylase 1 (LSD1) is a key epigenetic eraser enzyme implicated in cancer metastases and recurrence. Nuclear LSD1 phosphorylated at serine 111 (nLSD1p) has been shown to be critical for the development of breast cancer stem cells. Here we show that circulating tumor cells isolated from immunotherapy-resistant metastatic melanoma patients express higher levels of nLSD1p compared to responders, which is associated with co-expression of stem-like, mesenchymal genes. Targeting nLSD1p with selective nLSD1 inhibitors better inhibits the stem-like mesenchymal signature than traditional FAD-specific LSD1 catalytic inhibitors such as GSK2879552. We also demonstrate that nLSD1p is enriched in PD-1^+^CD8^+^ T cells from resistant melanoma patients and 4T1 immunotherapy-resistant mice. Targeting the LSD1p nuclear axis induces IFN-γ/TNF-α-expressing CD8^+^ T cell infiltration into the tumors of 4T1 immunotherapy-resistant mice, which is further augmented by combined immunotherapy. Underpinning these observations, nLSD1p is regulated by the key T cell exhaustion transcription factor EOMES in dysfunctional CD8^+^ T cells. EOMES co-exists with nLSD1p in PD-1^+^CD8^+^ T cells in resistant patients, and nLSD1p regulates EOMES nuclear dynamics via demethylation/acetylation switching of critical EOMES residues. Using novel antibodies to target these post-translational modifications, we show that EOMES demethylation/acetylation is reciprocally expressed in resistant and responder patients. Overall, we show for the first time that dual inhibition of metastatic cancer cells and re-invigoration of the immune system requires LSD1 inhibitors that target the nLSD1p axis.

## Introduction

T cell dysfunction is a hallmark of many cancers. Antibodies targeting the programmed cell death protein 1 (PD-1)/programmed death-ligand 1 (PD-L1) axis have shown impressive clinical efficacy in multiple cancer types (1, 2). PD-1 immunotherapy partially reinvigorates dysfunctional PD-1^+^ T cells, also known as exhausted T cells, though durable reinvigoration remains problematic and intrinsic and acquired resistance are common (2). The peripheral blood of cancer patients, particularly those with a high disease burden, is enriched for CD8^+^ T cells expressing checkpoint proteins indicating exhaustion and immunotherapy resistance, including PD-1, TIM3, TIGIT, and LAG3 (3–7). Circulating CD8^+^ T cells are highly heterogeneous both in cancer and chronic infection and often fail to express effector cytokines such as interferon (IFN)-γ (2, 5). Depending on the specific disease, including cancer and chronic viral infections, the circulating CD8^+^PD-1^+^ T cell population comprises 20–70% of total CD8^+^ T cells in hepatocellular, gastric, and lung cancer patients (4, 7, 8), while in solid tumors the CD8^+^PD-1^+^ T cell population can constitute as much as 80–90% of the resident CD8^+^ T cell population (8). There is an association between PD-1^hi^CD8^+^ T cell expression and increased disease severity in cancer patients (8), with a similar pattern is seen in lymphocytic choriomeningitis virus (LCMV), hepatitis C virus (HCV), and human immunodeficiency virus (HIV) infected patients (9–11).

Eomesodermin (EOMES) and T-bet are exhaustion and effector transcription factors (TFs) that also define two principal exhausted T cell subsets with different sensitivity profiles: EOMES^low^T-bet^high^PD-1^intermediate^ T cells can be re-invigorated by PD-1 blockade, whereas EOMES^high^T-bet^low^PD-1^high^ T cells cannot (5, 9, 12). High EOMES levels have consistently been linked with CD8^+^ T cell exhaustion in a variety of cancers and chronic viral infections (8–12). In contrast to this, complete elimination of EOMES was recently shown to inhibit CD8^+^ T cell effector function (12). However, the molecular mechanisms underlying EOMES regulation and T cell effector function and exhaustion are unknown.

Lysine-specific demethylase 1 (LSD1) is an H3K4 and H3K9 demethylase that also targets non-histone proteins including p53 (13) DNMT1 (14), and STAT3 (15). LSD1 is highly expressed in many aggressive cancer types including esophageal, squamous cell, hepatocellular, prostate, and basal-like breast cancers (16). LSD1 expression also increases through cancer evolution, and LSD1 expression has been shown to increase as pre-invasive ductal carcinoma *in situ* transitions to invasive ductal carcinoma (17). High LSD1 expression has also been associated with poor overall survival in patients with aggressive cancer (18). While anti-LSD1 therapies have recently been tested in the clinical setting, in solid tumors their efficacy is limited (19, 20). We recently showed that LSD1 is an important mediator of pro-EMT signatures in breast cancer stem cells (CSCs) and that LSD1 is induced in the CSC epigenome but not non-CSCs (21). Importantly, we also showed that nuclear LSD1 (nLSD1) expression is an important biomarker of poor patient prognosis. Phosphorylated nLSD1 (nLSD1p) enrichment in CSCs was mediated by protein kinase C (PKC), and nLSD1p was essential for CSC formation and recurrence (21).

Epigenetic programming plays a central role in the regulation of a variety of T cell subsets. Recently, LSD1 inhibition was shown to augment CD8^+^ T cell infiltration into tumors, suppressing tumor burden via enhanced chemokine expression (6) and by inducing endogenous retroviral elements leading to the activation of a type 1 IFN signature, which stimulated anti-tumor T cell immune function (22). We also recently showed that nLSD1 in complex with CoREST promotes immunosuppressive macrophage polarization in triple-negative breast cancer (TNBC) (23).

nLSD1p is therefore critical for CSC formation and cancer evolution. Here we show that nLSD1p and stem-like mesenchymal markers are increased in circulating tumor cells (CTCs) isolated from immunotherapy-resistant compared to responding metastatic melanoma patients. Targeting nLSD1p with nuclear axis LSD1 inhibitors better inhibits the stem-like mesenchymal signature than traditional FAD-specific LSD1 catalytic inhibitors (e.g., GSK2879552). We also demonstrate that nLSD1p is enriched in immune-exhausted T cells from treatment-resistant melanoma patients and in immunotherapy-resistant TNBCs *in vivo*. Furthermore, we show that targeting the LSD1p nuclear axis induces IFN-γ- and TNF-α-expressing CD8^+^ T cell infiltration into the tumor microenvironment in immunotherapy-resistant mice. We show for the first time that nLSD1p regulates the EOMES TF in dysfunctional CD8^+^ T cells, co-existing with EOMES in PD-1^+^CD8^+^ T cells in immunotherapy-resistant patients to regulate EOMES nuclear dynamics via demethylation/acetylation switching of key EOMES resides. The EOMES switch shows reciprocal expression in resistant and responding patients. Overall, these findings show for the first time that dual inhibition using epigenetic inhibitors that specifically target the nLSD1p axis in combination with immunotherapy is essential for the effective treatment of metastatic cancer.

## Materials and Methods

### Animal Studies

Five-week-old female BALB/c or BALB/c nude mice were obtained from the Animal Resources Center (ARC), Perth, and allowed to acclimatize for 1 week in the containment suites at The John Curtin School of Medical Research (JCSMR). All experimental procedures were performed in accordance with the guidelines and regulations approved by the Australian National University Animal Experimentation Ethics Committee (ANU AEEC). For the 4T1 syngeneic TNBC breast cancer mouse model, which displays either complete or partial resistance to immunotherapy based on the immunotherapy drug utilized (24, 25), BALB/c mice were shaved at the site of inoculation the day before subcutaneous injection into the right mammary gland with 2 × 10^5^ 4T1 TNBC cells resuspended in phosphate-buffered saline (PBS). For the MDA-MB-231 model, BALB/c nude mice were inoculated into the right mammary gland with 2 × 10^6^ MDA-MB-231 TNBC cells resuspended in PBS:Matrigel. Treatments were started on day 9 (4T1) or day 15 (MDA-MB-231) after inoculation when tumors reached approximately 50 mm^3^. Tumors were measured using external calipers and calculated using a modified ellipsoidal formula ½ (a/b^2^), where a = longest diameter and b = shortest diameter. In the 4T1 model, mice were treated with Abraxane (30 mg/kg), anti-PD-1 (10 mg/kg) every 5 days (two doses) and phenelzine (40 mg/kg) or EPI-111 (various doses) daily. In the MDA-MB-231 model, mice were treated with Abraxane (60 mg/kg) or docetaxel (10 mg/kg) weekly (three doses). All treatments were given intraperitoneally in PBS except for docetaxel, which was administered in DMSO. Tumors were collected on day 15 (4T1) or day 35 (MDA-MB-231) after treatments for flow cytometry, NanoString analysis, and CD8^+^ T cell enrichment for immunofluorescence microscopy.

### Human Liquid Biopsy Processing

CD8^+^ T cells were isolated from healthy donors or metastatic cancer patients [melanoma or metastatic breast cancer (MBC)]. Liquid biopsies were collected from ER^+^PR^+^HER2^−^ or triple-negative breast cancer (TNBC) stage IV MBC patients who had received any form of systemic therapy or melanoma patient cohorts as described below. Disease burden was assessed by standard of care monitoring [CT scans (RECIST 1.1), blood work, clinical symptoms/judgement]. Melanoma patients were selected for this biomarker study and classified into either responder or resistant groups based on response to immunotherapy using the RECIST 1.1 analysis as described in (26). Patient cohorts we also further divided into complete response (CR), partial response (PR), stable disease (SD), and progressive disease (PD). CR to SD were considered responders and PD resistant.

Mesenchymal circulating tumor cells (CTCs) were enriched using CD45 depletion from liquid biopsies of stage IV metastatic melanoma patients undergoing either nivolumab, ipilimumab, or pembrolizumab monotherapy or, if resistant to monotherapy, nivolumab, and ipilimumab dual therapy. Liquid biopsies were processed as previously (18, 21). Liquid biopsies were pre-enriched using the RosetteSep™ method to isolate CD8^+^ T cells by employing the RosetteSep™ Human CD8 enrichment Kit (15063, Stemcell Technologies, Vancouver, Canada) to remove CD45^+^ cells and red blood cells using density gradient centrifugation with SepMate™-15 (IVD) density gradient tubes (85420, Stemcell Technologies) and Lymphoprep™ density gradient medium (07861, Stemcell Technologies). All experimental procedures relating to human studies were performed in accordance with the guidelines and regulations approved by the ACT Health Research Ethics and Governance Office (Ethics ID ETH.11.15.217 and Ethics ID ETH.5.16.073). Written informed consent was received from all patients prior to inclusion in the study.

### Tissue Processing and Staining

Formalin-fixed, paraffin-embedded primary melanoma tumor biopsies were processed in the BondRX for OPAL staining (Perkin-Elmer, Waltham, MA) using the ER2 instrument protocol for 20 min at 100°C with Epitope Retrieval Solution (pH 6 EDTA-based retrieval solution) followed by probing with rabbit anti-EOMES-Ac, anti-EOMES-Me2, or pan-cytokeratin (Ab9377; Abcam, Cambridge, UK) and mouse host CD8 (provided by the Opal kit) antibodies and visualized with Opal kit 520, 570, 650, and 690. Coverslips were mounted on glass microscope slides with ProLong Clear Antifade reagent (Life Technologies, Carlsbad, CA). The 7-color Automation kit (NEL801001KT), 7-color Immune Discovery kit (OP7DS2001KT), 4-color Automation kit (NEL820001KT), and Opal 4 Lymphocyte kit (OP4LY2001KT) were used.

### Immunofluorescence Staining

Immunofluorescence microscopy was performed to determine the mean total nuclear fluorescence intensity (TNFI), the total cytoplasmic fluorescence intensity (TCFI), and Pearson's correlation coefficient (PCC) as previously described (21) PCC values were determined by the strength of the relationship between two fluorochrome signals. Primary antibodies were as detailed in the Supplementary Methods.

### Immunofluorescence Microscopy

Protein targets were localized by confocal laser scanning microscopy. Single 0.5 μm sections were obtained using a Leica DMI8 microscope running LAX software using a 100x oil immersion lens. The final image was obtained by averaging four sequential images of the same section. Digital images were analyzed using ImageJ software (ImageJ, NIH, Bethesda, MD) to determine either the TNFI, TCFI, or the nuclear/cytoplasmic fluorescence ratio (Fn/c) using the equation: Fn/c = (Fn– Fb)/(Fc – Fb), where Fn is nuclear fluorescence, Fc is cytoplasmic fluorescence, and Fb is background fluorescence. ImageJ software with automatic thresholding and manual selection of regions of interest (ROIs) specific for cell nuclei was used to calculate the PCC for each pair of antibodies. PCC values range from −1 = inverse of co-localization to +1 = perfect co-localization, with 0 = no co-localization. The Mann-Whitney non-parametric test (GraphPad Prism, GraphPad Software, San Diego, CA) was used to determine significant differences between datasets. Additionally, the Plot-Profile feature of Fiji-ImageJ was used to record the fluorescence intensity of a pair of antibody targets along a line through selected cell nuclei. For each dataset, at least 3 nuclear sections were counted for 3 separate cells and plotted with the mean ± SEM.

### High-Throughput LEICA DMI8 Population Microscopy

A Leica SP8 equipped with white light laser and UV 405 nm laser and SMD-HyD detectors and Leica LAS X software was used. Tile-scanned images were captured using the Leica LAS X Navigator module, field of view scanner, and sequential line scanning with a 63 × 1.4 oil immersion objective at 0.75 zoom, giving a pixel size of 0.48 μm. The final image was created by overlaying the 4 channels and stitching each tile to produce a single mosaic image. The mosaic image was used to determine the TNFI or TFI. The total number of cells was counted in a defined area using an automated stage and LAX analysis software to automatically select cells and measure fluorescent intensities. Resulting data were then employed to calculate T cell population dynamics expressed as a % of total cell population.

### ASI Digital Pathology System

Touching cells were automatically segmented, signal expression was quantitatively measured, and results per cell and over the entire scanned region displayed. For high-throughput microscopy, protein targets were localized by confocal laser scanning microscopy. Single 0.5 μm sections were obtained using an Olympus-ASI automated microscope with either a 20x lens or a 100x oil immersion lens running ASI software. The final image was obtained by employing a high-throughput automated stage with ASI spectral capture software. Digital images were analyzed using automated ASI software (Applied Spectral Imaging, Carlsbad, CA) to automatically determine the distribution and intensities with automatic thresholding and background correction of either the mean TNFI, TCFI, or TFI as well as the percentage population of cells expressing the analyzed proteins.

### Custom Antibodies

Custom polyclonal rabbit EOMES-641k-Ac, EOMES-641k-Me2, and EOMES-373k-Me2 were generated by Mimotopes^TM^ as detailed in the Supplementary Methods.

### Plasmid Constructs

The EOMES canonical sequence was examined and significantly conserved motifs for methylation and acetylation surrounding lysine 641 identified. This region overlapped/encompassed a putative nuclear localization sequence (NLS) domain. Three plasmid constructs were made: a canonical sequence and two mutants, mutant 1 mimicking an (alanine substitution) unmethylated and unacetylated state (mutant 1: lysine to alanine) and mutant 2 mimicking a non-acetylated, hyper-methylated state (lysine to phenylalanine) to test the importance of this motif on nuclear localization. Transfections were carried out using the NEON^TM^ Transfection System kit (MPK5000; Invitrogen) to transfect target cells with 15 μg of plasmid.

For LSD1, two plasmids were constructed, wild-type LSD1 (LSD1-WT) and the other containing a mutation at threonine 110 and serine 111 to alanine (LSD1-Mut) to prevent phosphorylation at this site and activation of the NLS (21). Transfections were carried out using the NEON^TM^ Transfection System kit (MPK5000; Invitrogen) to transfect target cells with 15 μg of plasmid.

### Jurkat T Cell Culture

The Jurkat stimulation model was used as previously described (27). The human Jurkat T cell line (Clone E6-1, ATCC TIB-152) was cultured in complete 10% fetal bovine serum (FBS) RPMI medium (Gibco, Life Technologies, Carlsbad, CA). For inhibitor studies, cells were pre-treated with phenelzine or GSK.

### Immunoblot and Dot Blot Analysis

Immunoblot analysis was performed using primary rabbit anti-human EOMES (AB23345) or our custom primary rabbit EOMES PTM antibodies and secondary HRP-conjugated goat-anti-rabbit antibody on nuclear extracts isolated from Jurkat T cells either transfected with EOMES WT plasmids, EOMES mutant 1 plasmid, LSD1 WT plasmid, or LSD1 NLS mutant plasmid. Signals were detected with enhanced chemiluminescence reagents (Western Lightning ECL-Plus; Perkin-Elmer, NEL104001) and film exposure. Band intensity signals were normalized to the total protein transferred to the blot detected using the Quantitative Novex Reversible Protein Stain (Thermo Fisher Scientific, IB7710) and Image J analysis.

### Half-Way ChIP

Half-ChIP assays were performed according to the manufacturer's instructions (Upstate Biotechnology, Millipore) and as previously described for Jurkat T cells (27). Fixation was performed as detailed, and fixed chromatin was sonicated with an Ultrasonic processor (Qsonica, Newtown, CT) under optimized conditions to produce average DNA fragments of approximately 500 bp. Prior to antibody addition, samples were pre-cleared with salmon sperm DNA-protein A-agarose, and the soluble chromatin fraction was incubated overnight at 4°C with a primary antibody to LSD1p (ABE1462) and Protein A magnetic beads. The beads were washed and incubated with immunoblot loading buffer containing beta-mercaptoethanol at 95°C and analyzed as above (immunoblot analysis) with a primary EOMES antibody (AB23345).

### Tumor Dissociation Protocol

4T1 tumors were harvested in cold DMEM supplemented with 2.5% FCS before being finely cut using surgical scalpels and enzymatically dissociated using collagenase type 4 (Worthington Biochemical Corp., Lakewood, NJ) at a concentration of 1 mg collagenase/g of tumor at 37°C for 1 h. Dissociated cells were then passed through a 0.2 μm filter before downstream assays.

### Flow Cytometry

Cells isolated from the 4T1 tumor mouse model were stimulated with PMA/ionomycin for 4 h in the presence of brefeldin A. Cells were surfaced stained with CD45, CD3, CD8, and intracellular staining of IFN-γ in the presence of brefeldin A and analyzed by flow cytometry. Student's *t*-test was used to compare control vs. other groups (^*^p < 0.05, *n* = 3).

CD8^+^ T cells isolated from TNBC patients were untreated or treated with phenelzine for 10 h *in vitro*, followed by stimulation with PMA/CaI for 4 h in the presence of brefeldin A. Cells were then surface stained with CD8 (BV421, BioLegend), CCR7 (AF647, BioLegend), and CD45RA (BV605, BioLegend) and intracellularly stained with perforin (PerCP-Cy5.5, BioLegend) and IFN-γ (PE-Cy7, BioLegend), and t-distributed stochastic neighbor embedding (tSNE) analysis was performed via flow cytometry.

### RNA Extraction

Total RNA was extracted from cells using the RNeasy Micro kit (Qiagen, Hilden, Germany) according to the manufacturer's protocols. RNA was measured using the Qubit RNA HS Assay kit (Thermo Fisher Scientific).

### NanoString nCounter Assay

Single cell suspensions were magnetically labeled with anti-CD8 microbeads UltraPure (Miltenyi Biotec, Bergisch Gladbach, Germany) in MACS running buffer. CD8 T cells were then positively isolated using the autoMACS Pro Separator (Miltenyi Biotec) according the manufacturer's protocols. Enriched cells were then snap frozen and RNA isolated using the RNeasy Micro kit (Qiagen). Samples were analyzed using the NanoString platform according to the manufacturer's procedures. Briefly, 50 ng of RNA was hybridized with the multiplexed mouse PanCancer Immune Profiling Panel codeset for 18 h at 65°C. Samples were then loaded onto the chip via the nCounter prep station and data acquired using the nCounter Digital Analyzer. Data analysis was performed using nSolver Analysis Software. Background correction was made by subtracting the mean + 2SD from the raw counts obtained with negative controls. Counts for target genes were then normalized with the geometric mean of housekeeping genes selected as the most stable ones using the geNorm algorithm. Normalized data were log2 transformed for further analyses.

### RNA-Seq

Sequences were cleaned (Trimmomatic-0.36, TagDust), mapped to Hg38 [HISAT2 (28)], and transcripts quantified [-count exons -pc 3 Homer (29)]. Differential genes were identified using DeSeq2 (30) with a 0.1 false discovery rate (FDR) and treating the four samples (from two different donors, with repeat samples collected at different times) as replicates and pairing control and treated samples. Principal component analysis (PCA) was performed on quantile normalized [HOMER (29)] samples using the factoextra package in R. Enriched Gene Ontology/pathway analysis and extraction of promoter (−600 to +100 bp) and enhancer sequences (600 bp) were performed in HOMER (29). Enhancer regions were obtained from publicly available ATAC-seq data from CD8^+^ lymphocytes (31, 32) and linked to differential genes if they were within 50 kb of the gene transcription start site (TSS). CLOVER (*p* < 0.05) (33) was used to find motif enrichment using the JASPAR 2016 PWMs against backgrounds with matching GC content (for promoters) or all enhancers within 50 kb of a gene TSS (enhancers).

We examined the enrichment of the phenelzine gene signatures in publicly available expression profiles from GSE72752 (34), GSE24081 (35), GSE85947 (36), GSE60501 (37), GSE84105 (38), GSE26495 (39), GSE12589 (40), GSE24151 (41), and GSE23321 (42). Normalized, unlogged data was obtained from GEO and the nominal *p*-value, normalized enrichment score (NES), and leading edge genes for our genesets were calculated by Gene Set Enrichment Analysis [GSEA (43); Signal2Noise, weighted].

### Quantification and Statistical Analysis

All statistical comparisons between sample groups were calculated using the two-tailed non-parametric Mann-Whitney test (GraphPad Prism, San Diego, CA) unless otherwise indicated. Where applicable, statistical significance is denoted by ^*^P ≤ 0.05, ^**^P ≤ 0.005, ^***^P ≤ 0.0005, and ^****^P ≤ 0.0001. Data are expressed as mean ± SE.

## Results

### Targeting LSD1's Nuclear Activity Effectively Inhibits Cancer Cell Line Mesenchymal Marker Expression

**Figure 1:**
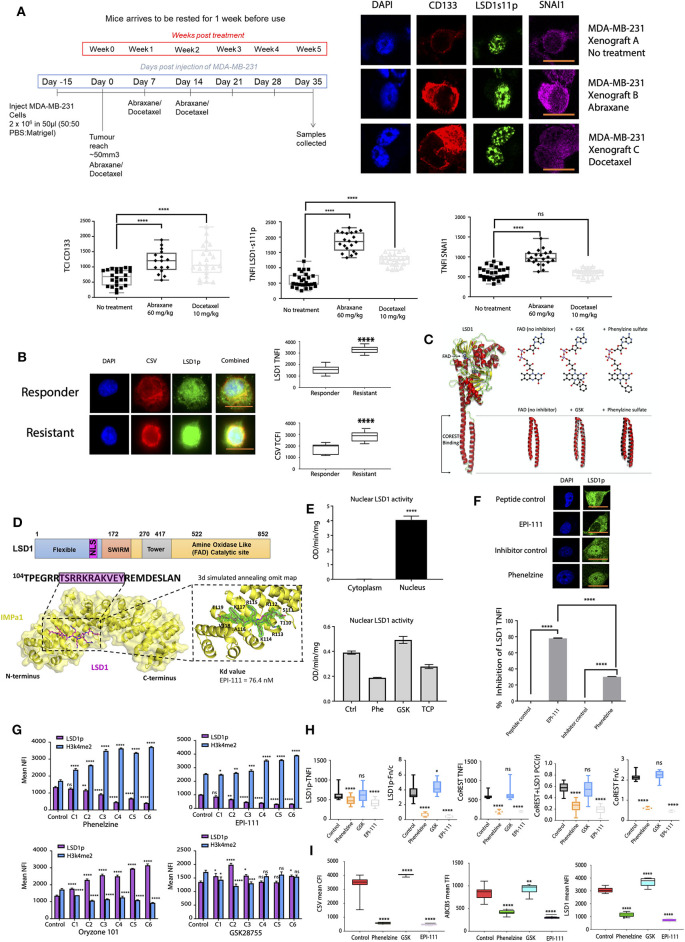
Targeting LSD1's nuclear activity effectively inhibits cancer cell line mesenchymal marker expression. **(A)** MDA-MB-231 cells were transplanted subcutaneously into the mammary fat pads of BALB/c nude mice and treated with intraperitoneal (IP) injections of vehicle control only, Abraxane (60 mg/kg), or docetaxel (10 mg/kg). Tumors were excised and digested into single cell suspensions and then surviving Abraxane-resistant tumor cells were subjected to immunofluorescence microscopy. The total nuclear fluorescence intensity (TNFI) was determined for control, Abraxane 60 mg/kg, and docetaxel 10 mg/kg treated tumors after probing with anti-CD133, anti-SNAI1, or LSD1-s111p antibodies (*n* > 10). All data represent the mean ± SE. *P < 0.05; **P < 0.01; ***P < 0.001; ****P < 0.0001, Mann-Whitney test. **(B)** CTCs were isolated from responder or resistant melanoma patient liquid biopsies, fixed, and labeled with primary antibodies targeting CSV and LSD1s111p. The TNFI for LSD1 and total cytoplasmic fluorescence intensity (TCFI) for CSV were measured using ImageJ (*n* > 20 cells per group, with *n* = 10 patients in the responder cohort and *n* = 10 patients in the resistant cohort; 4 samples per patient as in Supplementary Table 2). Graphs plot the TNFI of LSD1, TCFI of CSV. Mann-Whitney test, ****p < 0.0001, ***p = 0.0002, **p = 0.0021, *p = 0.033, ns > 0.05. **(C)** Structural superposition of LSD1 in the absence and presence of phenelzine and GSK. The structures solved in this study (left panel), LSD1 no inhibitor (left) (PDB 6NQM), LSD1:GSK (middle) (PDB 6NQU), and LSD1:phenelzine (right) (PDB 6NR5) are represented in cartoon mode. These structures are superimposed (left panel), showing a high degree of structural homology in the LSD1 catalytic domain for all three structures. LSD1 alone and LSD1:GSK also show high structural conservation in the alpha-helical tails; however, LSD1:phenelzine has a 5.4 Å displacement in this region. This region is important for CoREST binding, as shown in the middle panel (PDB 2UXX). Superposition of all structures in the left panel highlight that CoREST binding is mediated by the correct position of these domains. All images were generated in Pymol. **(D)** Protein structure of LSD1 showing key structural domains. Co-crystal structure of IMPα1 (below) (yellow cartoon and surface representation) and LSD1 NLS (stick representation, carbons in magenta); insert shows a 3σ simulated annealing omit map to support placement of LSD1 NLS residues. The binding affinity EPI-111 for IMPα2 is shown). **(E)** LSD1 activity (OD/min/mg) in nuclear and cytoplasmic extracts (5 μg protein/well) of MDA-MB-231 cells was measured using a H3K4me2 demethylase activity assay as described in the methods. Absorbance of LSD1 demethylated products was measured at 450 nm (with 655 nm reference filter) using a microplate spectrophotometer. Data are presented as the mean ± SEM and are representative of a single biological experiment performed in triplicate (*n* = 3). An unpaired parametric *t*-test was performed in GraphPad Prism to determine statistical significance. ****p ≤ 0.0001. LSD1 activity assay on nuclear extracts of RAW264.7 cells either untreated or treated with phenelzine, GSK, or LSD1 inhibitor tranylcypromine (below). **(F)** MDA-MB-231 cells were treated with control scrambled peptide, EPI-111, phenelzine, or GSK and labeled with primary antibodies targeting LSD1. The % inhibition of LSD1 TNFI was determined. IF analysis. Mann-Whitney test, ****p < 0.0001, ***p = 0.0002, **p = 0.0021, *p = 0.033, ns >0.05. **(G)** MDA-MB-231 cells were treated with phenelzine, GSK, EP-111, ORY-101, or control scrambled peptide, fixed, and labeled with primary antibodies targeting LSD1p and H34me2, and the TNFI values were measured using ImageJ (*n* > 20 cells/group). Graphs plot the TNFI of LSD1 and H3k4me2. Mann-Whitney test, ****p < 0.0001, ***p = 0.0002, **p = 0.0021, *p = 0.033, ns >0.05. **(H)** MDA-MB-231 cells were treated with phenelzine, GSK, EP-111, or control scrambled peptide, fixed, and labeled with primary antibodies targeting LSD1p and CoREST, and the TNFI values were measured using ImageJ (*n* > 20 cells/group). Graphs plot the TNFI of LSD1 and CoREST, Pearson's correlation coefficients (PCC) between LSD1 and CoREST, and the Fn/c (ratio of nuclear to cytoplasmic staining: below 1 is cytoplasmic biased, above 1 is nuclear biased). IF analysis. Mann-Whitney test, ****p < 0.0001, ***p = 0.0002, **p = 0.0021, *p = 0.033, ns >0.05. **(I)** MDA-MB-231 cells were treated with phenelzine, GSK, EP-111, or control, fixed, and labeled with primary antibodies targeting LSD1p, CSV, and ABCB5. The TNFI for LSD1, TCFI for CSV, and TFI for ABCB5 were measured using ImageJ (*n* > 20 cells/group). Graphs plot the TNFI of LSD1, TCFI of CSV, and TFI of ABCB5. Mann-Whitney test, ****p < 0.0001, ***p = 0.0002, **p = 0.0021, *p = 0.033, ns >0.05.

We recently showed that LSD1 phosphorylation at serine 111 (LSD1p) is critical for epithelial-to-mesenchymal transition (EMT) and is entirely nuclear (21). Consistent with our previous results in chemotherapy-resistant CTCs and MDA-MB-231 breast cancer cell lines (21), expression of nuclear LSD1p (nLSD1p) and other mesenchymal markers (SNAI1, CD133) was enriched in TNBC xenografts following treatment with Abraxane (nab-paclitaxel) and doxycycline (Figure 1A, Supplementary Figure 1A). We next examined nLSD1p expression in CTCs isolated from immunotherapy-resistant melanoma patients, with analysis revealing that CTCs were enriched for nLSD1p (Figure 1B).

nLSD1p co-exists with CoREST as a repressive complex, and CoREST is known to stabilize nLSD1p to demethylate LSD1's enzymatic targets H3K9 and H3K4 (44). We examined LSD1 using superimposed inhibitor-bound structures, which showed that phenelzine binding induces structural changes outside the catalytic region at the CoREST interaction domain on LSD1 (Figure 1C). This indicates that phenelzine has a dual role, targeting both the FAD and CoREST-binding domains of LSD1 [as we previously demonstrated in (23)], unlike GSK and ORYZON, which only target the FAD domain.

To address the ability of different LSD1 inhibitors to disrupt nLSD1p in metastatic cell lines, we compared the inhibitory effects of FAD domain (GSK and ORYZON), dual FAD/CoREST targeting inhibitors (phenelzine), and our novel reversible EPI-111 inhibitor (Patent no. WO 2018/045422 A1), a cell permeable peptidomimetic LSD1 inhibitor. EPI-111 has been designed and extensively optimized to selectively target the nuclear localization sequence (NLS) of LSD1 phosphorylated at serine 111, inhibiting interaction with importin a subunits and CoREST. EPI-111 displayed high binding affinity for importin α subunits (interaction at nanomolar concentrations to prevent nuclear translocation of LSD1) (Figure 1D, Supplementary Figures 1B–D). To determine the importance of nuclear localization on LSD1 activity, H3K4 demethylase activity was measured in nuclear and cytoplasmic extracts of human TNBC MDA-MB-231 cells. LSD1 H3K4 demethylation was only detected in the nuclear extracts of MDA-MB-231 cells and not in the cytoplasmic extracts (Figure 1E), confirming that H3K4 LSD1 activity is exclusively nuclear and that nuclear localization is essential for LSD1 demethylase activity in TNBC. *In vitro* phenelzine treatment reduced LSD1 nuclear enzymatic activity; however, GSK treatment did not inhibit LSD1 nuclear activity (Figure 1E), where the LSD1 inhibitor tranylcypromine (TCP) was used as a control.

To compare each LSD1 inhibitor in targeting nLSD1, immunofluorescence analysis was performed on MDA-MB-231 cells treated with phenelzine or EPI-111. EPI-111 showed ~80% inhibition of nLSD1 compared to ~30% for dual FAD/CoREST LSD1 inhibitors (Figure 1F). Furthermore, EP-111 was specific for LSD1 and had no effect on other epigenetic enzymes such as SETDB1 and EHMTZ2 (Supplementary Figure 1E).

Next, consistent with decreased nuclear LSD1 expression, phenelzine, EPI-111, but not GSK or ORYZON increased nuclear H3K4me2 expression in MDA-MB-231 cells and decreased nLSD1p expression (Figure 1G). Inhibition with phenelzine or EPI-111, but not GSK, inhibited nLSD1p and CoREST nuclear localization and colocalization in MDA-MB-231 cells and an immunotherapy-resistant melanoma (B16) cancer cell line (Figure 1H, Supplementary Figure 1F). Therefore, EPI-111 can disrupt the LSD1:CoREST complex, which destabilizes nLSD1p. Furthermore, the EMT markers CSV and ABCB5 were significantly reduced by phenelzine and EPI-111 in B16, 4T1, and MDA-MB-231 cells, whereas GSK induced their expression (Figure 1I, Supplementary Figure 1G). EPI-111 and phenelzine appear to disrupt the nuclear pool of LSD1 and more effectively reduce LSD1-regulated protein expression. A similar trend was also observed with respect to proliferation of several cancer cell lines (Supplementary Figures 2A,B).

In summary, FAD domain inhibitors such as GSK or ORYZON are unable to disrupt the nuclear LSD1p:CoREST complex in metastatic TNBC cell lines or disrupt the LSD1p and importin interaction. This indicates that importin-acting inhibitors and inhibitors that target the CoREST:LSD1 nuclear complex are superior to FAD domain only or dual acting inhibitors in reducing nLSD1p. These data demonstrate that, unlike the FAD-targeting inhibitors GSK or ORYZON, our optimized, cell permeable inhibitor EPI-111 can disrupt the LSD1-importin a subunit interaction as well as the interaction with CoREST and is specific for LSD1 (see Table 1).

**Table 1:** Comparison of the molecular effects of different LSD1 inhibitors.

	**EPI-111**	**Phenelzine sulfate**	**ORYZON**	**GSK**
FAD activity	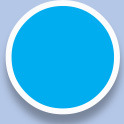	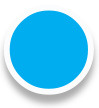	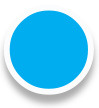	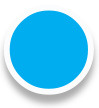
LSD1 nuclear pool specific (serine 111p)	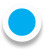	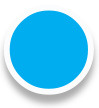	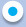	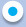
Blocks LDS1:importin alpha 1 & 3	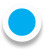	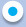	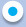	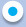
Targets NLS (phosphorylated at serine 111)	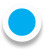	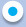	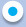	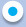
LSD1:CoREST destabilization	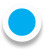	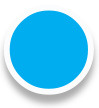	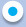	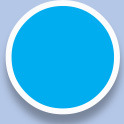

### Targeting LSD1's Nuclear Activity Effectively Inhibits Mesenchymal Marker Expression *in vivo*

**Figure 2:**
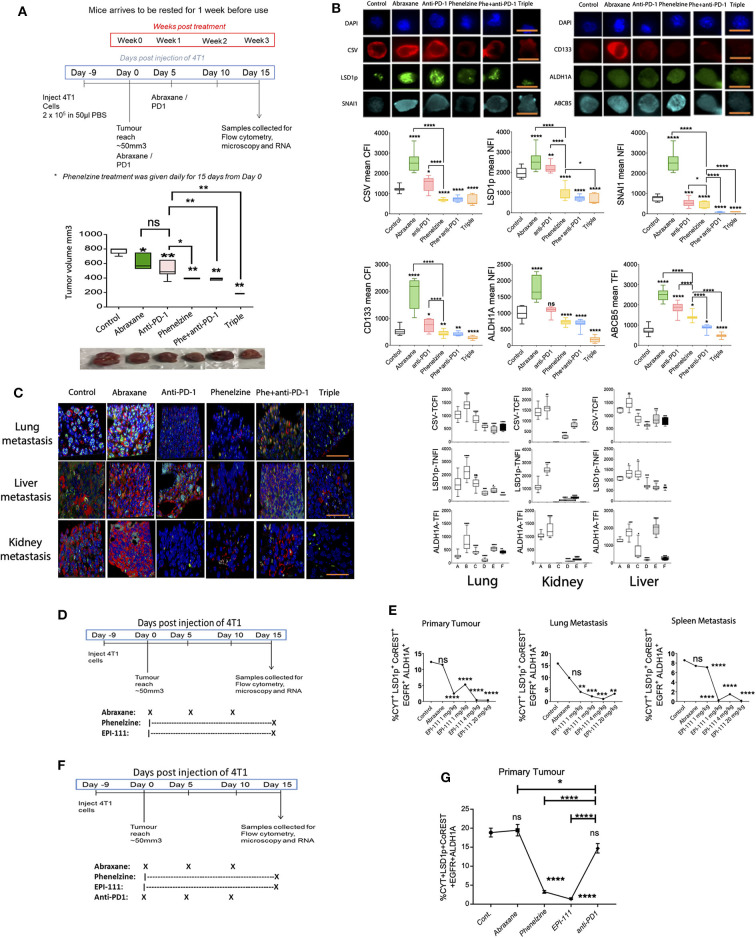
Targeting LSD1's nuclear activity effectively inhibits mesenchymal marker expression *in vivo*. **(A)** 4T1 breast cancer treatment regimen and tumor volumes on day 15 post-treatment. Mann-Whitney test, *p < 0.02, **p < 0.008. Treatment groups from left to right. Control, Abraxane (30 mg/kg), anti-PD-1 (10 mg/kg), phenelzine (40 mg/kg), phenelzine + anti-PD-1, and triple (Abraxane + phenelzine + anti-PD-1). **(B)** Cancer cells were isolated from a 4T1 TNBC immunotherapy-resistant mouse model treated as in 2A and labeled with primary antibodies against LSD1s111p, CSV, and SNAIL or CD133, ALDH1A, and ABCB5. The TNFI for LSD1, SNAIL, and ALDH1A, TCFI for CSV and CD133, and the TFI for ABCB5 were measured using ImageJ (*n* > 20 cells/group). Graphs plot the TNFI of LSD1, SNAIL, ALDH1A; TCFI of CSV, CD133; and TFI of ABCB5. Mann-Whitney test, ****p < 0.0001, ***p = 0.0002, **p = 0.0021, *p = 0.033, ns > 0.05. Representative images are shown with scale bar equal to 10 mm. Group A = control, Group B = abraxane, Group C = anti-PD1, Group D = phenelzine, Group E = phenelzine + anti-PD-1, and Group F = triple (PD-1 + phenelzine + abraxane). **(C)** FFPE sections were taken from primary tumors and lung, liver, and kidney metastases from a 4T1 TNBC immunotherapy-resistant mouse model treated as in 2A and labeled with primary antibodies against LSD1s111p, CSV, and ALDH1A. The TNFI for LSD1 and ALDH1A and TCFI for CSV were measured using ImageJ (*n* > 20 cells/group). Graphs plot the TNFI of LSD1, ALDH1A and TCFI of CSV. Mann-Whitney test, ****p < 0.0001, ***p = 0.0002, **p = 0.0021, *p = 0.033, ns > 0.05. Representative images are shown with scale bar equal to 30 mm. Group A = control, Group B = abraxane, Group C = anti-PD1, Group D = phenelzine, Group E = phenelzine + anti-PD-1, and Group F = triple (PD-1 + phenelzine + Abraxane). **(D)** 4T1 breast cancer treatment regimen. Treatment groups from left to right. Control, Abraxane (30 mg/kg), phenelzine (40 mg/kg), EPI-111 (1 mg/kg), EPI-111 (4 mg/kg), and EPI-111 (20 mg/kg). **(E)** FFPE sections were taken from primary tumors and lung and spleen metastases from a 4T1 TNBC immunotherapy-resistant mouse model treated as in 2D and labeled with primary antibodies against LSD1s111p, cytokeratin (CYT), CoREST, EGFR, and ALDH1A. The cancer cell population positive for these markers was analyzed using the ASI digital pathology system to enumerate % total cell population. Graphs plot the % cell population for each group (*N* > 500 cells a group). Mann-Whitney test, ****p < 0.0001, ***p = 0.0002, **p = 0.0021, *p = 0.033, ns > 0.05. Treatment groups are control, abraxane (30 mg/kg), phenelzine (40 mg/kg), EPI-111 (1 mg/kg), EPI-111 (4 mg/kg), and EPI-111 (20 mg/kg), with 5 mice per group. **(F)** 4T1 breast cancer treatment regimen. Treatment groups from left to right: control, abraxane (30 mg/kg), phenelzine (40 mg/kg), EPI-111 (4 mg/kg), and anti-PD1 (10 mg/kg), with 5 mice per group. **(G)** FFPE sections were taken from primary tumors from the 4T1 TNBC immunotherapy-resistant mouse model treated as in 2D and labeled with primary antibodies against cytokeratin, LSD1p, CoREST, EGFR, and ALDH1A. The cancer cells positive for these markers were analyzed using the ASI Digital Pathology System to enumerate % total cell population. Graphs plot the % cell population for each group (*n*>500 cells per group; *n* = 5 mice per group). Mann-Whitney test, ****p < 0.0001, ***p = 0.0002, **p = 0.0021, *p = 0.033, ns > 0.05.

LSD1p is therefore upregulated in chemoresistant cancer cell lines and in the CTCs of immunotherapy-resistant patients, and targeting nuclear LSD1p inhibits the mesenchymal, stem-like signature (Figures 1A,B,H,I). We next examined nLSD1p and mesenchymal marker expression in a 4T1 TNBC mouse model treated with phenelzine, anti-PD-1, or Abraxane either alone or in combination (PD1 and phenelzine) or as triple therapy (Figure 2A). Consistent with previous findings, anti-PD1 alone displayed a moderate reduction in tumor burden (24). Phenelzine alone was superior to anti-PD1 or Abraxane monotherapy in reducing overall tumor burden (Figure 2A). Phenelzine combined with immunotherapy as a double therapy or Abraxane and immunotherapy as a triple therapy further reduced primary tumor burden (Figure 2A). Using fluorescence microscopy to examine which tumor epithelial component was most affected by the single and combination therapies, Abraxane induced expression of mesenchymal (CSV, LSD1p, and ALHD1A) and stem-like markers (CD133, ALDH1A, and ABCB5) in the primary tumors consistent with our previous data (45), while phenelzine and its combinations reduced expression of these markers significantly more than anti-PD1 or Abraxane (which increased expression of these markers) (Figure 2B).

Next, mesenchymal markers (CSV, ALDH1A, and LSD1p) were examined in 4T1 lung, kidney, and liver metastases (Figure 2C). Anti-PD-1 variably altered mesenchymal marker expression in primary tumors and lung, liver, and kidney metastases (Figure 2C). Phenelzine and its combinations showed the most significant inhibition, with triple therapy showing greater overall inhibition. LSD1 inhibition not only inhibited tumor growth but specifically targeted those elements of the tumor that promote progression and therapeutic resistance, i.e., mesenchymal and stem-like cells and metastatic disease (Figures 2B,C). We next examined the effect of the nuclear LSD1p inhibitor EPI-111 in the 4T1 TNBC mouse cancer model (Figure 2D). In the tumor microenvironment, digital pathology analysis revealed that EPI-111 reduced the mesenchymal, stem-like population in both primary tumors and lung and spleen metastases. Phenelzine had a similar but lesser effect, and Abraxane did not significantly reduce expression of these markers (Figure 2E). In a 4T1 TNBC mouse cancer model, phenelzine or EPI-111 reduced the mesenchymal, stem-like population more than either anti-PD1 therapy or Abraxane, both of which did not significantly after the mesenchymal, stem-like population (Figures 2F,G).

### LSD1 Inhibition Re-invigorates CD8^+^ T Cell Subsets in Mouse Models

**Figure 3:**
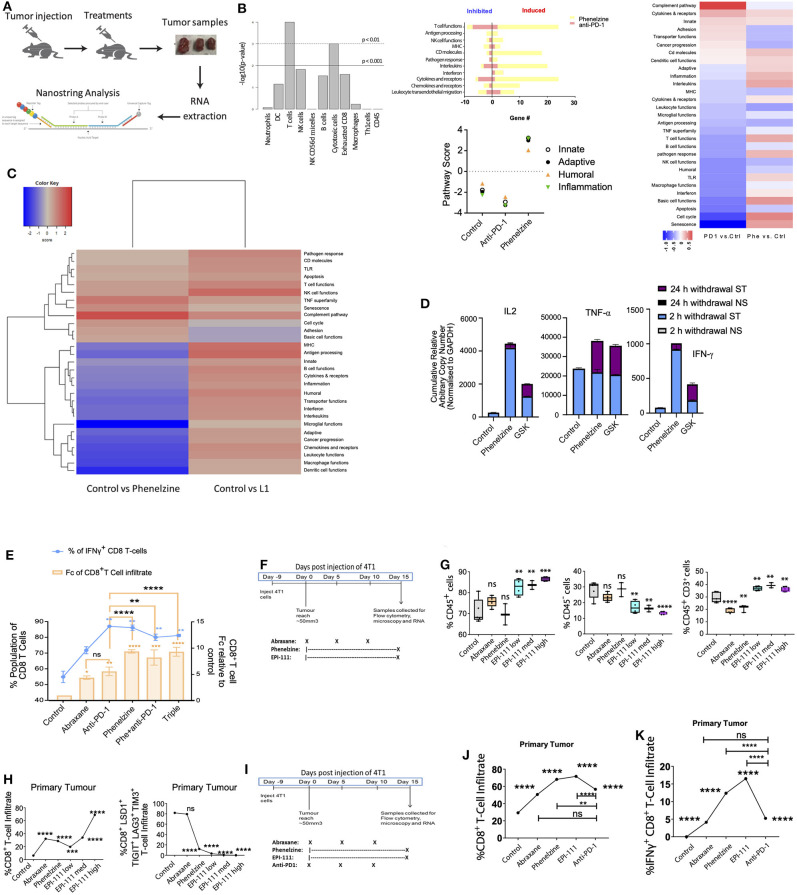
LSD1 inhibition re-invigorates CD8^+^ T cell subsets in mouse models. **(A)** Schematic of the metastatic breast cancer mouse model. Tumor samples from 3 individual animals/group were collected on day 15, RNA was extracted, and gene expression measured using the nanostring pancancer immune profiling panel. **(B)** Bar chart of -log10 transformed *p*-values across cell types by nCounter advanced analysis. Characteristic genes of various immune cell populations measure each population's abundance within the tumor sample. Immune pathway scores against treatment conditions defined using the first principal component of each geneset's data and each sample's gene expression profile condensed into a small set of pathway scores including innate, adaptive, humoral, and inflammation pathways. Total number of induced and inhibited genes for immune-related pathways analyzed by nanostring. Statistically significantly differentially expressed genes are defined by 2-fold linear changes with *p* < 0.05 compared to control samples. Yellow bar plot represents phenelzine, red bar plot represents anti-PD-1. **(C)** 4T1 tumor-bearing mice were treated with vehicle control, phenelzine (40 mg/kg), or EPI-111 (20 mg/kg). Fifteen days post-treatment, the primary tumors were measured and harvested and collected for nanostring analysis. NanoString global significance scores for RNA isolated from whole tumors. Heatmap displaying each sample's directed global significance scores. Directed global significance statistics measure the extent to which a geneset's genes are up- or down-regulated with the variable. Red denotes genesets whose genes exhibit extensive over-expression with the covariate, blue denotes genesets with extensive under-expression. NanoString analysis was performed in triplicate. **(D)** Jurkat T cells were treated with phenelzine or GSK for 10 h followed by inhibition withdrawal and resting for different time points. After resting for 24 h, cells were re-inhibited with phenelzine or GSK followed by repeat inhibition (PMA/CaI for 2 h), withdrawal, and resting. IFN-γ expression levels were measured by RT-PCR and normalized to GAPDH. Data represent the fold changes in expression of stimulated samples compared to control non-stimulated samples. Expression values are the average of the RT-PCR (technical) replicates, and error bars indicate min-max. **(E)** T cells isolated from 4T1 tumors were stimulated with PMA/ionomycin for 4 h in the presence of brefeldin A, stained for IFN-γ, and analyzed by flow cytometry (*p < 0.05, *n* = 3). Data overlies total CD8^+^ T cell infiltration into the primary tumor (*n* = 5 mice per group). Treatment groups are control, abraxane (30 mg/kg), anti-PD1 (10 mg/kg), phenelzine (40 mg/kg), anti-PD1 and phenelzine, or triple therapy. **(F)** 4T1 breast cancer treatment regimen. Treatment groups from left to right: Control, Abraxane (30 mg/kg), phenelzine (40 mg/kg), EPI-111 (1 mg/kg), EPI-111 (4 mg/kg), and EPI-111 (20 mg/kg), with 5 mice per group. **(G)** Cells were taken for FACS analysis from primary tumors from a 4T1 TNBC immunotherapy-resistant mouse model treated as in 3E and labeled with primary antibodies against CD45 and CD3 positive cells. Graphs plot the % cell population for each group (*n* = 5 mice per group). Mann-Whitney test, ****p < 0.0001, ***p = 0.0002, **p = 0.0021, *p = 0.033, ns > 0.05. **(H)** FFPE sections were taken from primary tumors from a 4T1 TNBC immunotherapy-resistant mouse model treated as in 2D and labeled with primary antibodies against CD8, LSD1, TIGIT, LAG3, and TIM3. The CD8^+^ T cell population positive for these markers was analyzed using the ASI digital pathology system to enumerate % total cell population. Graphs plot the % cell population for either CD8^+^ T cell infiltration or CD8^+^LSD1^+^TIGIT^+^LAG3^+^TIM3^+^ T cells (*N* > 500 cells per group, 5 mice per group). Mann-Whitney test, ****p < 0.0001, ***p = 0.0002, **p = 0.0021, *p = 0.033, ns > 0.05. Treatment groups are control, abraxane (30 mg/kg), phenelzine (40 mg/kg), EPI-111 (1 mg/kg), EPI-111 (4 mg/kg), and EPI-111 (20 mg/kg), with 5 mice per group. **(I)** 4T1 breast cancer treatment regimen. Treatment groups from left to right. Control, abraxane (30 mg/kg), phenelzine (40 mg/kg), EPI-111 (4 mg/kg), and anti-PD1 (10 mg/kg). **(J)** FFPE sections were taken from primary tumors from a 4T1 TNBC immunotherapy-resistant mouse model treated as in 2D and labeled with primary antibodies against CD8. The CD8^+^ T cell population within the FFPE section was analyzed using the ASI digital pathology system to enumerate % total cell population. Graphs plot the % cell population for either CD8^+^ T cell infiltration (*N* > 500 cells per group, *n* = 5 mice per group). Mann-Whitney test, ****p < 0.0001, ***p = 0.0002, **p = 0.0021, *p = 0.033, ns > 0.05. **(K)** FFPE sections were taken from primary tumors from a 4T1 TNBC immunotherapy-resistant mouse model treated as in 3J and labeled with primary antibodies against CD8 and IFN-γ. The CD8^+^ T cell population positive for both of these markers was analyzed using the ASI digital pathology system to enumerate % total double-positive cell population. Graphs plot the % cell population for CD8^+^IFN-g^+^ T cells (*n* > 500 cells per group; *n* = 5 mice per group). Mann-Whitney test, ****p < 0.0001, ***p = 0.0002, **p = 0.0021, *p = 0.033, ns > 0.05.

Qin, Vasilatos (6) demonstrated that LSD1 inhibition enhances T cell-attracting chemokine expression, thereby improving anti-tumor immunity when combined with checkpoint protein inhibition. We therefore wanted to understand the effect on CD8^+^ T cells of inhibiting nLSD1. We reasoned that since nLSD1p inhibition reduces mesenchymal stem-like markers and induces CD8^+^ T cell infiltration, this would enhance anti-tumor immune responses. Using the 4T1 metastatic TNBC model, we employed NanoString analysis to examine cellular and gene expression changes in primary tumors treated with phenelzine or anti-PD-1 therapy (Figure 3A). T cells (expressing *Cd2, Cd3e, Cd3g*, and *Cd6*) were the most abundant immune cell type in tumor tissues, followed by cytotoxic cells (expressing *Gzma* and *Klrd1*) (Figure 3B). In treated tumors, only phenelzine stimulated innate, adaptive, humoral, and general inflammatory responses as determined by immune pathway analysis (Figure 3B). When total induced and inhibited genes were profiled for enrichment of immune-related pathways, phenelzine significantly induced all immune function-related pathways, with only a few genes inhibited compared to controls. In particular, phenelzine upregulated T cell function, cytokines and receptors, interleukins, and CD molecules (Figure 3B) Conversely, anti-PD-1 treatment had a greater inhibitory effect on gene expression, especially T cell function (Figure 3B).

Next, to determine the effect of targeting nLSD1 on immune pathway upregulation, phenelzine- and EPI-111-treated 4T1 TNBC tumors were compared using the NanoString platform. Immune pathway analysis showed that EPI-111 resulted in overexpression of almost all immune-related pathways relative to controls (Figure 3C). In contrast to phenelzine, EPI-111 upregulated the expression of MHC and antigen processing pathways. Our data extend the effects observed by Qin, Vasilatos (6) that nLSD1p inhibition with phenelzine increases expression of CD8^+^ T cell-related immune effector genes. Furthermore, our data show that specifically targeting nLSD1 with EPI-111 further upregulates immune pathways in solid tumors.

Overall, phenelzine had a potent immune stimulatory effect in the tumor microenvironment consistent with CD8^+^ T cell activation and reinvigoration. Next, we employed a Jurkat T cell transcriptional memory model [Jurkat Tm; (12, 46)] to investigate the effect of nLSD1p inhibition on T cell transcriptional memory (Figure 3D). Phenelzine, but not GSK, dramatically increased IFN-γ gene expression at all resting time points (Figure 3D), suggesting that phenelzine but not GSK enhances transcriptional memory in CD8^+^ T cells even after inhibition withdrawal through inhibition of LSD1's catalytic and nuclear activities. In agreement with Qin, Vasilatos (6), in the mouse 4T1 TNBC model, while Abraxane and PD-1 treatment increased the infiltrating CD8^+^ T cell population phenelzine alone or in combination had a superior effect in inducing CD8^+^ T cell infiltration, and particularly CD8^+^IFN-γ^+^ T cell infiltration (Figure 3E). A similar trend of increased cytokine expression in CD8^+^ T cells was also observed in the 4T1 TNBC mouse cancer model (Supplementary Figure 3A).

### nLSD1p Inhibition Induces Immune Cell Infiltration and Re-invigoration and Enhances Tm in Cd8^+^ T Cells in Mouse Models and Humans With Metastatic Breast Cancer

We reasoned that since LSD1 inhibition reduces mesenchymal stem-like markers and induces CD8^+^ T cell infiltration, targeting nLSD1p directly with EPI-111 would enhance anti-tumor immune responses. We therefore examined the effect of EPI-111 in the mouse 4T1 TNBC model (Figure 3F). In the tumor microenvironment, EPI-111 increased CD45^+^ and CD3^+^CD45^+^ T cell infiltration in a dose-dependent manner while reducing the CD45^−^ population (Figure 3G). Targeting nLSD1p with EPI-111 also increased CD8^+^ T cell infiltration as well as reducing checkpoint markers for exhausted CD8^+^ T cells (Figure 3H).

We next examined the effect of monotherapy with EPI-111, phenelzine, anti-PD-1, or Abraxane in the 4T1 TNBC mouse model (Figure 3I). Monotherapy with EPI-111 or phenelzine significantly increased total CD8^+^ T cells, which was superior to both anti-PD1 and Abraxane treatment (Figure 3J). We next examined the effect of EPI-111, phenelzine, anti-PD-1, or Abraxane on total IFN-γ^+^CD8^+^ T cell infiltration (double positive for CD8 and IFN-γ). Phenelzine or EPI-111 also significantly increased total IFN-γ^+^CD8^+^ T cell infiltration over that of anti-PD1 or Abraxane (Figure 3K). This is in line with our previously described data showing that phenelzine or EPI-111 more effectively reduce the mesenchymal, stem-like population within cancer and the overall tumor burden (Figures 2A,B,E,G).

**Figure 4:**
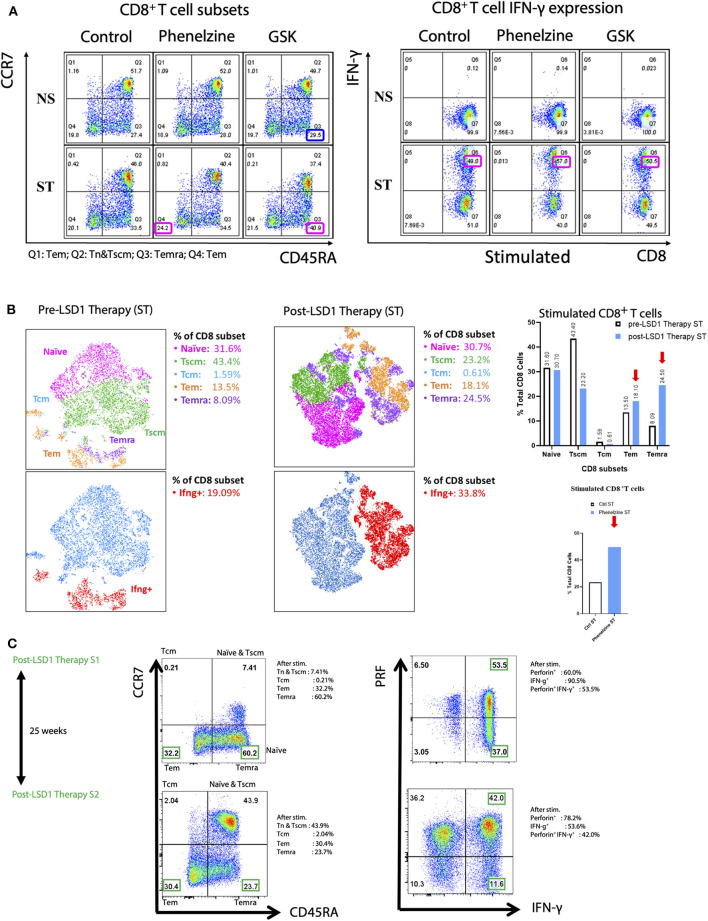
nLSD1p inhibition induces immune cell infiltration and re-invigoration and enhances Tm in CD8^+^ T cells in mouse models and humans. **(A)** CD8^+^ T cells from TNBC patients were untreated or treated with phenelzine, GSK, or control *in vitro* and stained with CCR7 and CD45RA antibodies to categorize them into naïve, T effector memory (Tem), and T effector memory RA (Temra). Cells were also stained with perforin and IFN-γ after stimulation with PMA/CaI for 4 h in the presence of brefeldin A. **(B)** t-distributed stochastic neighbor embedding (tSNE) analysis was performed on patient-derived TNBC CD8^+^ T cells either pre or post LSD1 therapy after PMA/ionomycin stimulation. Gated populations were divided into Naïve, Tscm, Tcm, Tem, and Temra phenotypes. **(C)** tSNE analysis was performed on patient-derived TNBC CD8^+^ T cells post LSD1 therapy or post 25 weeks LSD1 therapy after PMA/ionomycin stimulation. Gated populations (naïve, Tem, and Temra) of distinct phenotypes were overlaid onto a 2-dimensinal tSNE data space, revealing the differential expression of IFN-γ and perforin in total CD8^+^ T cells or different CD8 subsets.

Having addressed the importance of nLSD1p in the 4T1 TNBC mouse model, we next investigated the role of nLSD1p inhibition on memory T cell (Tm) subsets in human breast cancer liquid biopsies. Several studies have demonstrated the importance of memory T cell subsets in immunotherapy responses in cancer and viral immunity (1, 34, 47), so we examined their distribution in CD8^+^ T cells derived from a stage IV metastatic breast cancer patient treated with paclitaxel/trastuzumab/pertuzumab followed by *in vitro* phenelzine treatment. Flow cytometry analysis of the CD8^+^ T cells showed that phenelzine inhibition increased the Tem (CD45RA^−^CCR7^−^; effector memory) population from 20.1 to 24.2%, the Temra (CD45RA^+^CCR7^−^; effector memory re-expressing) population (48) from 33.5 to 34.%, and induced total CD8^+^IFN-γ expression from 49 to 50%. In comparison, GSK increased Temra from 33.5 to 40.9% and GSK had no effect on Tem (Figure 4A). In line with this, treatment of CD8^+^ T cells isolated from a metastatic stage IV TNBC patient analyzed by digital pathology revealed that phenelzine treatment significantly increased protein expression of Ki67 and IFN-γ (Supplementary Figure 3B). A similar trend was observed in CD8^+^ T cells treated with phenelzine *ex vivo* derived from a stage IV ER/PR^+^/HER2 metastatic breast cancer patient (Supplementary Figure 3C) and a progressive disease (PD) melanoma patient (Supplementary Figure 3D).

We next examined memory T cell subsets before and after anti-LSD1 therapy in a metastatic breast cancer patient receiving LSD1 inhibitor therapy in combination with standard of care. Flow cytometry analysis revealed that, post-therapy, Tem increased from 13.5 to 18.1% and Temra from 8.09 to 24.5%. Total CD8^+^IFNγ^+^ T cells also increased from 19.09 to 33.8% (Figure 4B) following T cell activation (Supplementary Figure 3E).

We also examined the memory T cell population after a significant interval of 25 weeks after LSD1 therapy ended. Tem decreased from 32.2 to 30.4% and Temra decreased from 60.2 to 23.7%. Overall, IFN-γ^+^ cells decreased from 90.5 to 53.6%; however, perforin^+^CD8^+^ T cells increased from 60 to 78.2% and double-positive perforin^+^IFN-γ^+^ CD8^+^ T cells decreased from 53.5 to 42% (Figure 4C). Overall, after withdrawal of LSD1 therapy, re-programming of CD8^+^ T cells from metastatic breast cancer patient liquid biopsies persists.

### LSD1 Inhibition Promotes Conserved Effector Gene Expression Signatures in Cd8^+^ T Cells From Cancer Patients

**Figure 5:**
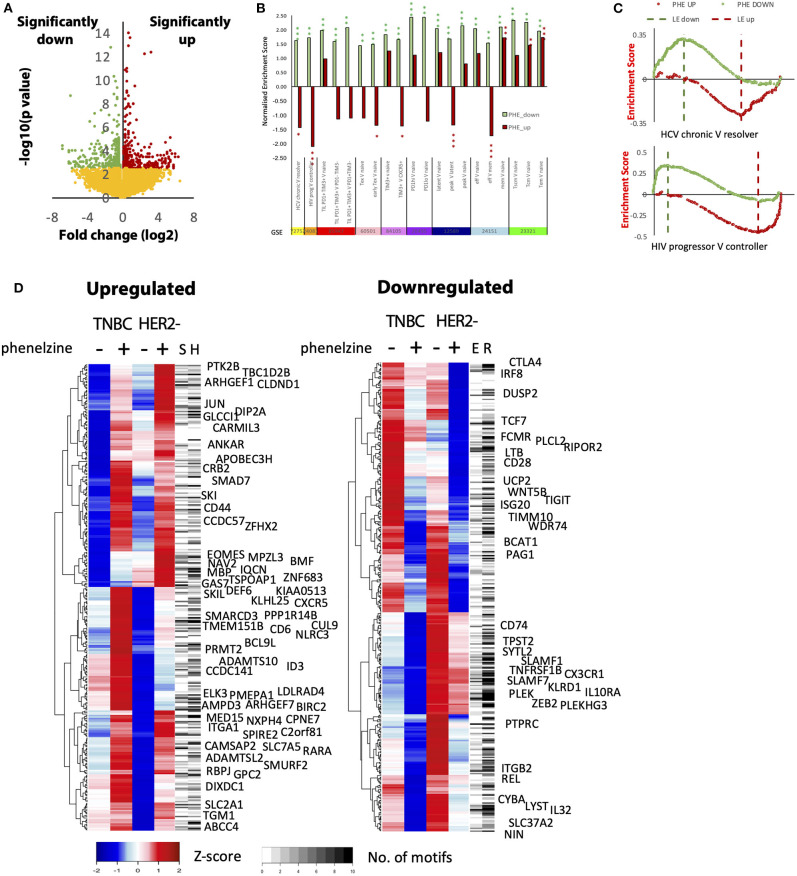
Global transcriptome analysis shows that phenelzine-mediated gene signatures are common to reinvigoration phenotypes in different model systems. **(A)** Volcano plot of the difference in gene expression before and after phenelzine treatment in TNBC and HER2^−^ donor CD8^+^ T cell transcriptomes. Significance was determined in DeSeq2, FDR < 0.1, *n* = 4. Three hundred fifty genes were downregulated with phenelzine (PHE) treatment and 314 were upregulated. **(B)** Enrichment of the down (PHE_Down) and up (PHE_UP) PHE signatures in different models of T cell exhaustion, activation, and memory. For x vs. y comparisons, a positive normalized enrichment score (NSE) indicates enrichment in x, a negative score enrichment in y. *p < 0.05 **p < 0.01 ***p < 0.001. **(C)** Enrichment plots for GSE72752 and GSE24081 showing individual genes from the two phenelzine signatures distributed across the ranking in expression from chronic to resolver or progressor to controller. **(D)** Expression profiles of the genes up- and downregulated by phenelzine in the two donors (TNBC and HER2^−^). Averages of the duplicate isolations and treatments are shown (*n* = 2). The number of SMAD (S) and HIC2 (H) or EOMES (E) and RUNX1 (R) motifs in nearby enhancers are marked. Upregulated genes with at least 5 SMAD motifs in nearby enhancers or that are negative regulators of the TGF-b pathway are named. Downregulated genes with at least 5 EOMES motifs in nearby enhancers are named (red: direct (bound) EOMES targets upregulated by EOMES overexpression; blue: direct (bound) targets downregulated by EOMES overexpression).

To determine how LSD1 inhibition with phenelzine globally impacts gene expression programs, CD8^+^ cells isolated from liquid biopsies from TNBC and HER2^−^ breast cancer patients were treated with phenelzine and subjected to RNA-seq. Phenelzine upregulated 314 genes and downregulated 350 genes (FDR 0.1; Figure 5A). When compared with published exhausted CD8^+^ T cell datasets by gene set enrichment analysis (GSEA), genes upregulated by phenelzine were significantly (p < 0.05) enriched in resolver (34) (HCV) and controller (35) (HIV) infected patients; in naïve compared to early exhausted T cells; CXCR5^+^ compared to TIM3^−^ T cells (38); latent compared to peak infections (40); memory compared to effector (41); and memory compared to naïve (42) cells (Figure 5B). Genes downregulated by phenelzine were significantly (*p* < 0.05) enriched in chronic (34) (HCV) and progressor (35) (HIV) infected patients; exhausted compared to naive or activated T cells (36, 37, 39); TIM3^+^ compared to naïve or CXCR5^+^ (38); peak compared to latent (40) infections; and effector or memory compared to naïve cells (41, 42) (Figures 5B,C). Upregulated genes were significantly enriched for the microtubule cytoskeleton and the TGFβ receptor signaling pathway, the latter mainly representing negative regulators such as *SMURF2, SMAD7, SKI*, and *SKIL* (Figure 5D). Downregulated genes were significantly enriched for immune, cell surface signaling, and locomotion pathways (Figure 5D, Supplementary Table 1) and were leading edge genes in non-resolving infections or the exhausted state (Supplementary Figures 4A,B). Furthermore, both phenelzine and GSK significantly inhibited the expression of exhaustion markers (*PD1, CTLA4, TIM3*, and *TIGIT*) in CD8^+^ T cells from HIV-1 infected and treated patients (data not shown). With respect to transcription factor motifs in the promoters of differentially expressed genes, upregulated genes were enriched for homeodomains and downregulated genes for zinc fingers, RUNX, NFAT, and forkheads (Supplementary Figure 4B). As many factors bind away from promoter regions, we identified putative enhancers using ATAC-seq data from naïve, effector, memory, and exhausted CD8^+^ T cells (31, 32). Analysis of open regions near (< 50 kb) upregulated genes showed enrichment of the SMAD2:SMAD3:SMAD4 and HIC2 motifs and members of the ETS family, while those of the downregulated genes were enriched for the RUNX1, THAP1, PPARG::RXRA, and T-box families (Supplementary Figure 4B. Upregulated genes with multiple SMAD motifs included *CD44*, while downregulated genes with multiple EOMES motifs included *TIGIT* and *TNFRSF1B* (Figure 5D, Supplementary Figure 4B). Overall, targeting the nuclear axis of LSD1:CoREST with phenelzine induced transcriptional programs in cancer T cells consistent with reinvigorated T cell programs conserved in other model systems.

### The EOMES:LSD1p Nuclear Complex Is Enriched in PD-1^+^CD8^+^ T Cells From Resistant, High Disease Burden Patients

**Figure 6:**
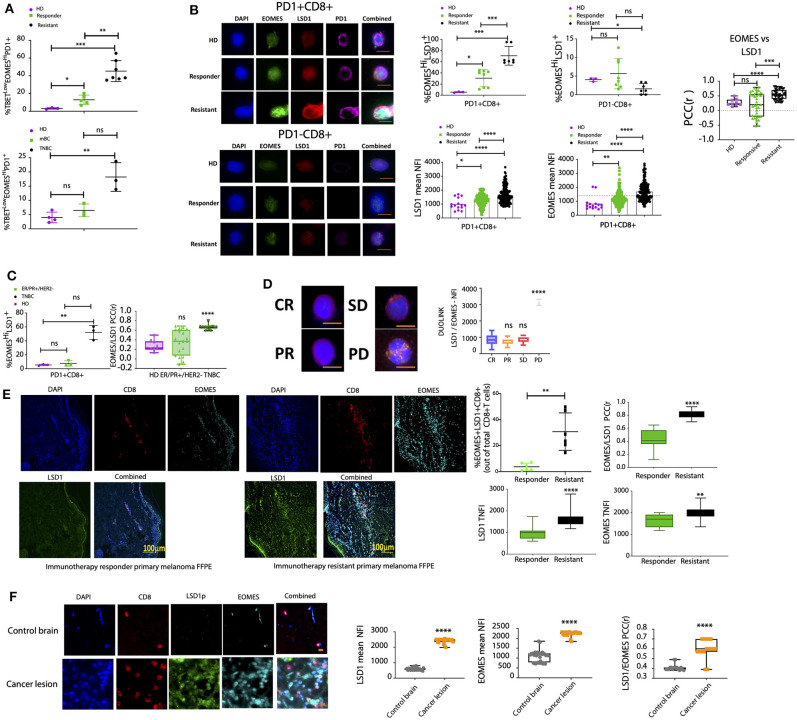
The EOMES:LSD1p nuclear complex is enriched in PD-1^+^CD8^+^ T cells from resistant, high disease burden patients. **(A)** CD8^+^ T cells were isolated from healthy donors, melanoma patient cohorts, or metastatic breast cancer patient (ER^+^/PR^+^/HER2- or TNBC) liquid biopsies and labeled with primary antibodies targeting T-bet, EOMES, and PD-1. ASI Digital Pathology Analysis was carried out to calculate the percentage population of T-bet^Low^EOMES^Hi^PD-1^+^ T cells. Patient cohorts included *n* = 5 healthy donors, *n* = 20 patients in the responder cohort, and *n* = 15 patients in the resistant cohort, with sampling as in Supplemental Table 2 with 4 samples per patient. **(B)** Primary antibodies against LSD1 and EOMES were used to label PD-1^+^CD8^+^ T cells from healthy donors or melanoma patients with different immunotherapy susceptibility profiles. Samples were processed by ASI Digital Pathology Analysis based on >500 cells (patient cohorts included *n* = 5 healthy donors, *n* = 20 patients in the responder cohort, and *n* = 15 patients in the resistant cohort, with sampling as in Supplemental Table 2 with 4 samples per patient). The % EOMES^Hi^LSD1^+^PD-1^+^CD8^+^ T cell population was analyzed for each group as well as the TNFI of LSD1 and EOMES. Representative images for each dataset are shown with scale bar = 10 mm. Graphs represent either the % cell population (positive for EOMES/LSD1) or the TNFI for LSD1 and EOMES measured using digital pathology (ASI) minus background (*n* > 500 cells/patient sample for 40 patient samples). The EOMES:LSD1 PCC was determined for at least 40 individual cells ± SEM. Mann-Whitney test, ****p < 0.0001, ***p = 0.0002, **p = 0.0021, *p = 0.033, ns *p* > 0.05. **(C)** Primary antibodies against LSD1 and EOMES were used to label PD-1^+^CD8^+^ T cells from healthy donors or metastatic breast cancer patients (ER^+^/PR^+^/HER2^−^ or TNBC). Samples were processed by ASI digital pathology analysis based on >500 cells. Patient cohorts included *n* = 5 healthy donors, *n* = 20 patients in the responder cohort, and *n* = 15 patients in the resistant cohort, with sampling as in Supplemental Table 2 with 4 samples per patient. The % EOMES^Hi^LSD1^+^PD-1^+^CD8^+^ T cell population was analyzed for each group. Graphs represent the mean % positive cell population measured using digital pathology (ASI) minus background (*n* > 500 cells/patient sample for 40 patient samples). The EOMES:LSD1 PCC was determined for at least 40 individual cells ± SEM. Mann-Whitney test, ****p < 0.0001, ***p = 0.0002, **p = 0.0021, *p = 0.033, ns *p* > 0.05. **(D)** Proximity ligation assay (DuoLink) for EOMES and LSD1 in CD8^+^ T cells from melanoma patients with different immunotherapy susceptibility profiles. Representative images shown with scale bar = 10 mm. Graph plotted measures the PLA (ligation intensity measured by high-resolution microscopy) protein interaction. Patient cohorts = 3 patients per group and 4 repeat samples per patient. **(E)** Melanoma primary tumor baseline tissue biopsies for either responder or resistant melanoma patient cohorts were stained for LSD1, EOMES, and CD8 with DAPI. Representative images for each dataset are shown. Graphs represent either the % population of EOMES^+^LSD1^+^CD8^+^ T cells or the mean TNFI for LSD1 and EOMES measured using ASI Digital Pathology minus background (*n* > 50 cells/patient sample for 20 patient samples). The EOMES:LSD1 PCC was determined for at least 20 individual cells ± SEM. Mann-Whitney test, ****p < 0.0001, ***p = 0.0002, **p = 0.0021, *p = 0.033, ns *p* > 0.05. *n* = 20 patient samples total; *n* = 10 responder patient samples, *n* = 10 resistant patient samples (with 4 repeat samples per patient). **(F)** Metastatic brain cancer lesions from a metastatic breast cancer patient were stained for LSD1, EOMES, and CD8 with DAPI. Representative images for each dataset are shown. Graphs represent the mean TNFI for LSD1 and EOMES measured using ASI Digital Pathology minus background (*n* > 50 cells/patient sample for 30 patient samples). The EOMES:LSD1 PCC was determined for at least 20 individual cells ± SEM. Mann-Whitney test, ****p < 0.0001, ***p = 0.0002, **p = 0.0021, *p = 0.033, ns *p* > 0.05.

Previous studies have reported enrichment of PD1^+^ T cells in patient blood and tumor biopsies, with 30–70% of T cells positive for PD-1 (4, 7, 8). In addition, several studies have demonstrated that EOMES^high^T-Bet^low^ characterizes exhausted T cells and immunotherapy resistance (9, 49, 50), and similarly we observed a significantly higher proportion of CD8^+^ T cells positive for nuclear EOMES^high^ and significantly lower nuclear T-bet expression in PD-1^+^CD8^+^ T cells in immunotherapy-resistant melanoma patients (as defined by RECIST 1.1, Supplementary Figure 5A) and in PD-1^+^CD8^+^ T cells from TNBC patients (Figure 6A). PD-1^+^CD8^+^ T cells also had reduced expression of TNF-α, IFN-γ, and Ki67 compared to responder patients and were positive for checkpoint inhibitor proteins (TBET, LAG3, TIGIT) (Supplementary Figures 5B–E,G).

We therefore examined EOMES and LSD1p expression using digital pathology analysis in circulating PD-1^+^CD8^+^ T cells from cancer patients. Expression of nLSD1p and EOMES with PD-1 was enriched in CD8^+^ T cells from immunotherapy-resistant melanoma patients (Figure 6B). 63.4% (± 6.4%) of all CD8^+^ T cells were LSD1^+^EOMES^+^PD-1^+^ in resistant melanoma patients, compared to only 30% (± 5.4%) in responder patients and 5.5% (± 0.62%) in healthy donors (Figure 6B). Further, nuclear LSD1 and EOMES were co-expressed in the same cells and enriched in resistant patients (Figure 6B). Immunofluorescence co-localization analysis revealed high LSD1-EOMES correlations in resistant patients (Pearson's correlation coefficient (PCC) 0.6) and low correlations in responder and HD patients (PCC 0.28 and 0.12, respectively) (Figure 6B). Similarly, LSD1-EOMES nuclear co-expression and the LSD1^+^EOMES^+^PD-1^+^ profile were significantly higher in CD8^+^ T cells isolated from TNBC than ER^+^PR^+^HER2^−^ breast cancer patients (Figure 6C).

LSD1-EOMES co-expression was further examined by a proximity ligation assay, which only detects signal when the two target proteins are in a close proximity or complexed (51). LSD1:EOMES nuclear complexation was low in responders and high in immunotherapy-resistant patients (Figure 6D, Supplementary Figure 5F).

Similar to the pattern seen in circulating CD8^+^ T cells, CD8^+^ T cells examined by digital pathology in resistant melanoma tissue sections revealed a larger CD8^+^ T cell population positive for co-expression of nuclear LSD1 and EOMES than in responder melanomas (Figure 6E). A similar trend was observed in CD8^+^ T cells in metastatic breast cancer lesions within the brain, which expressed significantly higher levels of nuclear EOMES and LSD1, which were positively correlated as LSD1-EOMES nuclear complexes compared to normal brain lesions (Figure 6F).

### An EOMES Methylation/Acetylation Switch Regulated by LSD1 in CD8^+^ T Cells Indicates Immunotherapy Responsiveness

Our data suggest that an innate nuclear pool of LSD1 co-exists with EOMES as a nuclear complex in dysfunctional T cells in primary and secondary resistant metastatic melanoma patients and metastatic breast cancer patients. Further, nLSD1 release induces an effector function in dysfunctional CD8^+^ T cells. Given that EOMES plays a key role in inducing T cell exhaustion, we next addressed how LSD1 modulates EOMES in T cells.

**Figure 7:**
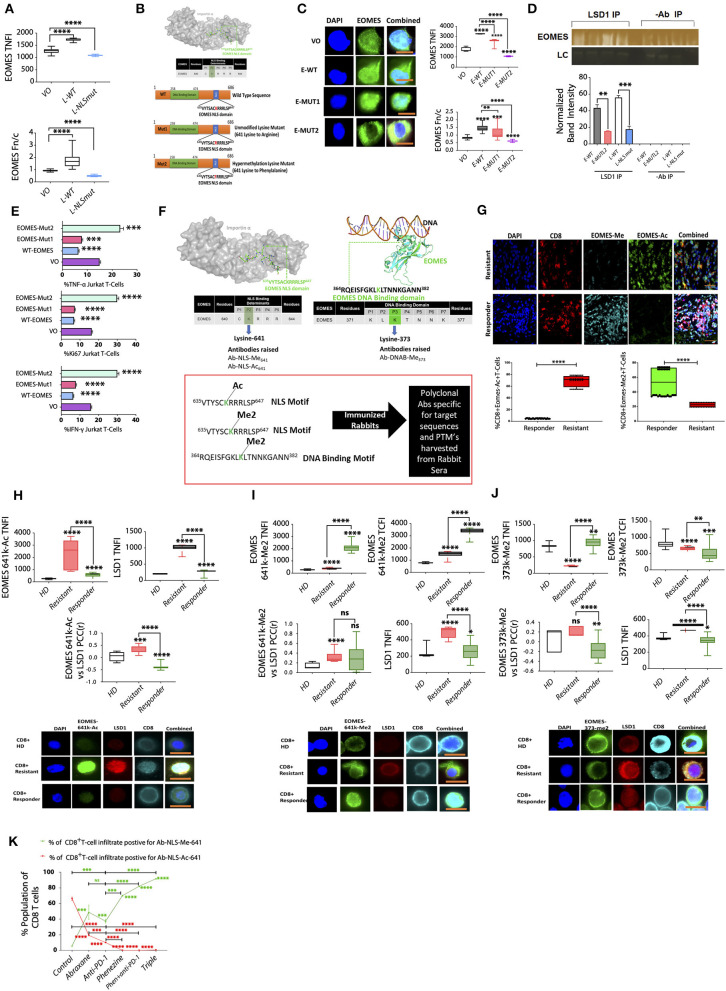
An EOMES methylation/acetylation switch regulated by LSD1 in CD8^+^ T cells indicates immunotherapy responsiveness. **(A)** Jurkat T cells transfected with either VO, LSD1 wildtype (LSD1-WT), or LSD1 NLS mutant plasmids (LSD1-NLSmut) were probed with primary antibodies targeting EOMES and screened by IF. Graphs represent the TNFI and Fn/c for *n* = 20 or more cells (*n* = 3 experiments). Mean NFI and Fn/c are shown. Mann-Whitney test, ****p < 0.0001, ***p = 0.0002, **p = 0.0021, ns >0.05. **(B)** Schematic of EOMES plasmids and 641k motif. **(C)** Jurkat T cells transfected with either VO, EOMES-WT, EOMES-Mut1, or EOMES-Mut2 plasmids and probed with primary antibodies targeting EOMES and screened by IF. Graphs represent the TNFI and Fn/c for *n* = 20 or more cells (*n* = 3 experiments). Representative images for each dataset are shown; scale bar indicates 10 μM. Mean NFI and Fn/c are shown. Mann-Whitney test, ****p < 0.0001, ***p = 0.0002, **p = 0.0021, ns >0.05. **(D)** Nuclear extracts from jurkat T cells transfected with LSD1 WT or mutant plasmids and subjected to half-way CHIP using LSD1 pull down or a no antibody control. Samples were subject to immunoblot analysis and probed with a primary rabbit antibody to human EOMES; representative bands are shown. EOMES band intensity was plotted using ImageJ software minus background for *n* = 3 with mean ± SEM. Group 1, EOMES WT; Group 2, EOMES-Mut2; Group 3, LSD1 WT; Group 4, LSD1 NLS mutant. **(E)** EOMES plasmids and Jurkat T cells transfected with either VO, EOMES-WT, EOMES-Mut1, or EOMES-Mut2 plasmids and probed with antibodies targeting Ki67, IFN-γ, and TNF-α. Graphs show the percentage of cells calculated from ≥ 500 cells in 3 experiments (*n* = 3) and the mean TFI of markers (*n* ≥ 20 cells). Representative images for each dataset are shown; scale bar indicates 10 μM. Mean TFI and the population % are shown. Mann-Whitney test, ****p < 0.0001, ***p = 0.0002, **p = 0.0021, ns >0.05. **(F)** EOMES protein structure showing the NLS and DNA-binding domain and the custom antibodies raised against specific PTMs. **(G)** Primary melanoma baseline biopsies classified as responder or resistant and processed for 3D high-resolution digital pathology with primary antibodies to CD8, EOMES-Ac, or EOMES-Me2 with DAPI. Representative images for each dataset are shown (*n* ≥ 90 cells/sample). The % population of each target is plotted along with scale bar = 15 mm. *n* = 20 patient samples total; *n* = 10 responder patient samples, *n* = 10 resistant patient samples. **(H)** CD8^+^ T cells were isolated from healthy donors (HD) and immunotherapy resistant or responsive melanoma patients and screened by IF microscopy for **(H)** EOMES-641k-Ac or **(I)** EOMES-641k-Me2 or **(J)** EOMES-373k-Me2 and LSD1. For each LSD1:EOMES pair, the PCC was determined (*n* = 10 patients, ≥20 cells/patient). Both the nuclear and cytoplasmic fluorescence intensity of each marker are plotted with significant differences indicated as well as the Fn/c; Mann-Whitney test, ****p < 0.0001, **p = 0.0021, ns>0.05. IF microscopy patient cohorts included *n* = 5 healthy donors, *n* = 15 patients in the responder cohort, and *n* = 15 patients in the resistant cohort, with sampling as in Supplemental Table 2 with 4 samples per patient. **(I)** CD8^+^ T cells were isolated from healthy donors (HD), immunotherapy resistant or responsive melanoma patients and screened by IF microscopy for EOMES-641k-Me2 and LSD1. For each LSD1:EOMES-641k-Me2 pair, the PCC was determined (*n* = 10 patients per group, ≥20 cells/patient). Both the nuclear and cytoplasmic fluorescence intensities of each marker are plotted with significant differences indicated as well as the Fn/c; Mann-Whitney test, ****p < 0.0001, **p = 0.0021, *p < 0.05, ns>0.05. IF microscopy patient cohorts included *n* = 5 healthy donors, *n* = 15 patients in the responder cohort, and *n* = 15 patients in the resistant cohort, with sampling as in Supplemental Table 2 with 4 samples per patient. **(J)** CD8^+^ T cells were isolated from healthy donors (HD) and immunotherapy resistant or responsive melanoma patients and screened by IF microscopy for EOMES-373k-Me2 and LSD1. For each LSD1:EOMES-373k-Me2 pair, the PCC was determined (*n* = 10 patients per group, ≥20 cells/patient). Both the nuclear and cytoplasmic fluorescence intensities of each marker are plotted with significant differences indicated as well as the Fn/c; Mann-Whitney test, ****p < 0.0001, **p = 0.0021, *p < 0.05, ns>0.05. IF microscopy patient cohorts included *n* = 5 healthy donors, *n* = 15 patients in the responder cohort, and *n* = 15 patients in the resistant cohort, with sampling as in Supplemental Table 2 with 4 samples per patient. **(K)** IF microscopy was performed on 4T1 syngeneic metastatic cancer model-derived CD8^+^ T cells fixed and probed with antibodies targeting EOMES-641k-Ac and CD8 or EOMES-641k-Me2 and CD8 with DAPI. >10,000 cells/group were scanned to profile the % positive population of infiltrating CD8^+^ T cells in the primary tumor microenvironment for each EOMES marker (*n* = 5 mice per group). Significant differences between groups are indicated as per the Mann-Whitney test, ****p < 0.0001, **p = 0.0021, ns>0.05.

To assess the importance of nLSD1p in EOMES nuclear dynamics, we constructed two LSD1 plasmids targeting serine 111 phosphorylation within LSD1's nuclear localization sequence (NLS), which is critical for LSD1 nuclear maintenance: a wild-type sequence, and an NLS mutant with a non-functional NLS (21) (Supplementary Figure 6A). We used Jurkat T cells with low endogenous LSD1 levels transfected with LSD1-WT and LSD1-NLS mutant plasmids. The transfected cells were then labeled with an EOMES antibody, and the nuclear:cytoplasmic ratio (Fn/c) and the total nuclear fluorescence intensity (TNFI) of EOMES analyzed. The TNFI of EOMES significantly increased with LSD1-WT transfection (TNFI of 1270 ± 22.57 to 1,735 ± 50.87), as did the Fn/c (0.9237 to 1.7, where an Fn/c < 1 is cytoplasm biased). In contrast, transfection with the LSD1-NLSmut resulted in a significant reduction of both the EOMES TNFI (TNFI of 1,093 ± 21.7) and a significant cytoplasmic bias with an Fn/c of 0.46 (Figure 7A).

Given that LSD1 regulates the nuclear dynamics of a variety of transcription factors such as p53 via demethylation (13) as well as N-terminal chromatin-associated post-translational modifications (PTMs), we wanted to establish what role LSD1 may have in regulating the nuclear dynamics of EOMES and if LSD1 demethylates any residues within any potential NLS motifs. Therefore, we examined whether EOMES contained any putative LSD1 demethylation targets. *In silico* analysis revealed a C-terminus sequence (^635^VYTSACKRRRLSP^647^) with a high probability of mediating nuclear dynamics and stabilization (Figure 7B), with lysine 641 identified as both an LSD1 methylation and acetylation target (52). We hypothesized that lysine 641 was likely to play a critical role in EOMES nuclear function and/or influence potential nuclear target specificity, so constructed three EOMES plasmids: E-WT containing wildtype EOMES; E-Mut1, with a lysine 641 to arginine mutation to mimic the unmethylated, unacetylated state; and E-Mut2, with a lysine 641 to phenylalanine mutation to mimic hypermethylation (Figure 7B). Transfection of these constructs into Jurkat T cells revealed that lysine 641 mutation to a hypermethylation mimic (E-Mut2) restricted expression to the cytoplasm, whereas E-WT and E-Mut1 showed nuclear bias, with the wild-type showing stronger nuclear expression than E-Mut1 (Figure 7C).

Next, halfway ChIP was used to examine the effect of the constructs on nuclear LSD1:EOMES interactions. Jurkat T cells were transfected with wild type LSD1 or EOMES plasmids, LSD1 mutant S-A(111) (biasing LSD1 to the cytoplasm), or EOMES-Mut-2 (mimicking permanent lysine 641 hypermethylation) (Figure 7D). Excluding LSD1 from the nucleus with the LSD1-S-A(111) mutant prevented EOMES pull-down in the half-way ChIP, and, similarly, when EOMES was hypermethylated, there was a significantly reduced interaction with LSD1 within the nucleus and consequently no EOMES pull-down (Figure 7D). Nuclear EOMES and LSD1 are therefore required for the expression of the LSD1:EOMES nuclear complex and LSD1 must be able to demethylate EOMES within the nucleus. These data also suggest that lysine 641 within the NLS is subject to regulation by LSD1 to influence the nuclear dynamics of EOMES.

Next, we examined the effect of nuclear EOMES overexpression in Jurkat T cells by transfection with the EOMES plasmids. Transfection with E-Mut1 or E-WT significantly reduced both the expression and proportion of TNF-α, IFN-γ, and Ki67 (Figure 7E) expressing cells, while E-Mut2 induced the expression and proportion of TNF-α, IFN-γ, and Ki67 expressing cells (Figure 7E). Demethylation of lysine 641 within the nuclei of Jurkat T cells appears to inhibit effector function and vice versa.

Next, we carried out further *in silico* analysis, which identified a second DNA-binding domain at lysine 373. To assess EOMES-DNA interactions, a homology model of an EOMES:DNA complex was generated based on the X-ray structure of the DNA-binding domain in T-bet, the 72% DNA-binding domain sequence identity between EOMES and T-Bet and overlay providing 100% confidence in the model. The conserved lysine 373 in EOMES is conserved in T-Bet (lysine 243), the latter shown to associate with DNA phosphate groups and methylation of which would be predicted to disrupt DNA binding (Figure 7F).

Therefore, to explore the function of both the lysine 641 and 373 residues, specific rabbit polyclonal antibodies were raised to lysine 641 for either methylation or acetylation and to lysine 373 residue for methylation (for the peptide sequences used please see Supplementary Table 3). Novel antibodies were required since the target motif is not covered by commercial antibodies; in particular, the Abcam EOMES antibody (AB23345) targets amino acid 650 to the C-terminus so does not target the residues regulating methylation/acetylation. Ten Rabbits were used to raise antibodies against 5 peptides (two rabbits per peptide) as detailed in the methods (see also the detailed methodology for antibody generation in the Supplementary Methods). The antibody raised against the unmodified motif was screened against both the target modified sequence (Supplementary Table 3 and Supplementary Methods; details of the negative controls, blanks, and example dilutions testing the specificity of each PTM antibody are detailed in Supplementary Tables 5–7) and the unmodified target sequence as an additional negative control antigen for each antibody target, with ELISA blank and negative controls included to account for background non-specific labeling. Antibodies raised against the unmodified NLS motif (641k) did not bind to the methylated or acetylated lysine 641 motif; conversely, the antibodies targeting the methylated or acetylated 641k motif did not bind to the unmodified motif and only their specific acetylated or methylated 641k motif. The same result was seen for the DNA-binding motif at lysine 373. Collectively, this validation demonstrates that these antibodies are specific only for the specific target sequence and are able to exquisitely discriminate the unmodified from the PTM sequences.

Next, we further studied the specificity using dot blot analysis, which revealed that the custom antibodies were not only specific for the intended sequence but also the specific PTM, with the 641-me2 antibody not targeting the acetylated peptide. Furthermore, treatment of the methylated peptide motif with LSD1 abrogated the dot blot signal, indicating PTM specificity (Supplementary Figure 6B; peptide sequences are in Supplementary Table 3). Immunoblot analysis of Jurkat T cells treated with phenelzine or EPI-111 demonstrated that, as expected, LSD1 inhibition induced EOMES-641-Me2 and reduced EOMES-641-Ac, consistent with our hypothesis that LSD1 mediates these PTMs (Supplementary Figure 6C; peptide sequences are in Supplementary Table 3). To further probe antibody specificity, Jurkat T cells were treated with blocking peptides specific for the antibody targets, further demonstrating that these antibodies are specific for the motif and PTM (Supplementary Figure 6D; peptide sequences are in Supplementary Table 3).

We next used fluorescence microscopy to examine lysine 641 methylation and acetylation in CD8^+^ T cells infiltrating immunotherapy responsive and resistant melanoma tissues prior to treatment. Analysis of the CD8^+^ T cell population revealed that EOMES lysine 641 was acetylated in 70% of CD8^+^ tumor-infiltrating lymphocytes in resistant patients vs. only 4.8% in responders, whereas the same motif was methylated in 54% of responders and 22.5% of resistant melanoma tissues (Figure 7G). We next assessed EOMES PTM expression by fluorescence microscopy in healthy donors and immunotherapy-responsive or -resistant melanoma patients. Resistant, non-responsive CD8^+^ T cells expressed low levels of cytoplasmic EOMES lysine 641 and 373 methylation. Nuclear EOMES-641-Ac expression was significantly higher in these resistant, non-responsive CD8^+^ T cells, with strong co-localization with LSD1 in resistant populations (Figure 7H) and correspondingly no nuclear EOMES-641-Ac in CD8^+^ T cells from responders. Strikingly, there was significant EOMES-641-Me2 or EOMES-373-Me2 expression in both the cytoplasm and nuclei of responder CD8^+^ T cells only. Methylated EOMES at 373 or 641 was expressed in the cytoplasm but not nuclei of resistant, non-responsive CD8^+^ T cells (Figures 7I,J). With regards to healthy donor (HD) CD8^+^ T cells, nuclear expression of EOMES-641-Ac was very low, similar to but significantly lower than responders. Interestingly, EOMES-641-Me2 was significantly higher in responder CD8^+^ T cells versus HD CD8^+^ T cells, whereas nuclear EOMES-373-Me2 was only modestly higher than HD samples (Figures 7H–J).

Finally, we employed digital pathology to examine the effect of Abraxane, anti-PD-1 treatment, or phenelzine on EOMES PTMs in the 4T1 TNBC model. Sequence analysis using BLAST indicated that the target peptide sequence of human and mouse EOMES is 91% identical, with only 1 amino acid difference (Supplementary Table 4). However, the target lysine at 641 is 100% preserved, as are the motifs and NLS. Anti-PD-1 treatment or Abraxane monotherapy significantly reduced EOMES 641 acetylation and increased methylation in infiltrating CD8^+^ T cells, while phenelzine even further decreased the proportion CD8^+^ T cells positive for nuclear EOMES-641-Ac and increased the proportion of CD8^+^ T cells positive for EOMES-641-Me2 (Figure 7K). Phenelzine alone or in combination increased T cell infiltration (Figure 7K). Strikingly, phenelzine eliminated almost all EOMES-641-Ac CD8^+^ T cells, with combination treatments having no additional efficacy. Similarly, compared to Abraxane or anti-PD-1 monotherapy, phenelzine induced a higher proportion of EOMES-641-Me-expressing CD8^+^ T cells, with combination therapies having even greater effects (Figure 7K).

## Discussion

This study extends our previous findings and discovers a dual role for nuclear LSD1 in the direct molecular regulation of CSCs, key seeders of metastasis, and dysfunctional T cells, a central feature of metastatic cancers, particularly immunotherapy-resistant cancers. We show for the first time that: (i) targeting the nuclear axis of LSD1 better inhibits CSC and mesenchymal signatures than traditional FAD-specific LSD1 catalytic inhibitors such as GSK2879552 (GSK); (ii) targeting nLSD1 reinvigorates CD8^+^ T cell subsets and enhances transcriptional memory in CD8^+^ T cells; and (iii) nLSD1p co-exists with the exhaustion transcription factor EOMES in PD-1^+^CD8^+^ T cells in immunotherapy-resistant patients and regulates EOMES nuclear dynamics via a demethylation/acetylation switch.

LSD1 is frequently expressed in aggressive diseases, and high levels of LSD1 are often associated with aggressive cancer phenotypes (18). In our previous work, we showed that phosphorylated nuclear LSD1 (phosphorylated at serine 111; nLSD1p) is enriched in breast cancer cell lines, chemotherapy-resistant MDA-MB-231 cancer xenografts *in vivo*, and CTCs isolated from patients with stage IV metastatic breast cancer (21). Other studies have also shown that LSD1 is implicated in regulating cancer stem cell signatures in glioblastomas (53) and hepatocellular carcinoma (54). We also previously demonstrated via genome-wide sequence analysis that nLDS1p promotes a mesenchymal and cancer stem cell-like transcriptional and epigenetic signature in breast cancer cells (21).

Here we found that nLSD1p expression was higher in CTCs isolated from liquid biopsies from immunotherapy-resistant melanoma patients. We also show that nLSD1p is enriched in immunotherapy-resistant cancer cells and is associated with an increase in a stem-like, mesenchymal signature in immunotherapy-resistant 4T1 TNBCs following treatment with anti-PD-1 immunotherapy or Abraxane. Our data indicate that the highly resistant, aggressive tumors enriched for nLSD1p are more resistant to chemo- or immunotherapy and are responsive to LSD1 inhibitors that exclusively target the nuclear LSD1 axis. We previously showed that the highly selective catalytic inhibitors GSK and ORYZON target LSD1 exclusively via the FAD domain (23); these catalytic LSD1 inhibitors have shown disappointing efficacy in the treatment of solid tumors such as metastatic melanoma, metastatic breast cancer, and Ewing sarcoma (55) due to targeting the FAD domain alone. Indeed, nuclear LSD1 has previously been shown to be critical for the maintenance of a stem-like signature and tumorigenicity of glioma stem cells and is associated with poorer patient prognosis (56–59).

This study and previous publications (21, 23) have shown that nLSD1p co-exists with CSC signatures in resistant cancer cells. Importantly, we found that targeting the nuclear axis of LSD1 with a novel LSD1 NLS peptidomimetic inhibitor, EPI-111, better inhibited nLSD1p and stem-like and mesenchymal signatures than other traditional FAD inhibitors. EPI-111 reduced the population of stem-like and mesenchymal cells to a greater extent than phenelzine and GSK in highly immunotherapy-resistant cancer cell lines. Our data also demonstrated that phenelzine or EP-111 reduced nLSD1p and the CSC-like, mesenchymal signature in primary tumors and metastatic lesions in an immunotherapy-resistant 4T1 mouse TNBC model. This is consistent with our previous results using siRNA to knock down LSD1 in breast cancer cell lines (21). We also showed that anti-nLSD1p therapy in combination with chemo- or immunotherapies further inhibited the CSC metastatic phenotype. These data highlight the importance of targeting nLSD1 outside the catalytic domain in solid tumors.

Targeting LSD1 in cancer models has previously been shown to elicit immune pathways that reprogram refractory tumors toward immunotherapy responsiveness (6, 22). The recent clue that LSD1 inhibition might indirectly modify T cell and immunotherapy responses through upregulation of chemokines and their receptors to enhance CD8^+^ T cell tumor infiltration prompted us to look more closely at this phenomenon (6, 22). Specifically, Qin, Vasilatos (6) demonstrated that LSD1 knockdown induced cytotoxic T cell-attracting chemokines and subsequent trafficking of CD8^+^ T cells into the tumor microenvironment, whereas Sheng, LaFleur (22) demonstrated that inhibition of LSD1 induced retroviral expression and enhanced tumor immunogenicity, increasing T cell infiltration. However, these studies did not address what direct effects LSD1 inhibition may have on T cells.

We show for the first time in the 4T1 TNBC model that targeting the nLSD1p:CoREST complex via EPI-111 increased CD45^+^ and CD3^+^CD45^+^ T cell infiltration in a dose-dependent manner while reducing the CD45^−^ population. Targeting the nuclear axis of LSD1p with EPI-111 in this model also increased CD8^+^ T cell infiltration and reduced checkpoint markers for exhausted CD8^+^ T cells. Monotherapy with EPI-111 or phenelzine significantly increased total CD8^+^ T cells and IFN-γ^+^CD8^+^ T cell infiltration, and this was more effective than both anti-PD1 and Abraxane therapies.

Furthermore, we showed that nLSD1 inhibition resulted in immune reinvigoration and the overexpression of key immune-related pathways, inducing T cell functions, antigen processing and pathogen responses, and significant T cell infiltration with a reduction in the checkpoint markers TIGIT, LAG3, and TIM3. Importantly, targeting the nuclear axis of LSD1 increased IFN-γ expression, and this was enhanced in combination with Abraxane in CD8^+^ T cells in 4T1 TNBCs.

We found that *ex vivo* nLSD1 treatment with phenelzine increased the population of effector memory CD8^+^ T cell subsets derived from a stage IV metastatic breast cancer patient who had withdrawn from phenelzine treatment, showing that anti-nLSD1 treatment induces a persistent effector memory response. This suggests for the first time an epigenetic memory response in TNBC patient CD8^+^ T cell subsets. We recommend that future large clinical studies should aim to determine optimal dosing strategies to sustain durable responses and effector memory maintenance. It is generally thought that LSD1 induces T cell infiltration by indirect mechanisms such as induction of T cell chemokines or through cancer cell-specific mechanisms. However, we have shown that nLSD1 inhibition also induces an IFN-γ response and is superior to catalytic inhibitors.

RNA sequencing data from CD8^+^ T cells from TNBC and HER2^−^ breast cancer patients treated with control or phenelzine were overlaid with global transcriptome signatures from exhausted T cells from HIV patients. Following treatment with phenelzine, CD8^+^ T cell signatures in metastatic breast cancer patients were the same as control HIV patients, suggesting that LSD1 inhibition might be able to re-invigorate exhausted immune cells in other chronic infections such as HIV.

LSD1 displays roles beyond histone demethylation by direct regulation of key transcription factors associated with CSC function (21). We have now demonstrated that nLSD1p co-exists with the exhaustion transcription factor EOMES and modulates EOMES nuclear translocation dynamics. Transfection with EOMES plasmids allowed us to detect acetylation and demethylation residues and show that nLSD1p demethylates and acetylates critical lysine residues within the NLS motif or DNA-binding motif of EOMES to restrict nuclear translocation.

However, we also now demonstrate that EOMES and nLSD1p are enriched in dysfunctional PD-1^+^CD8^+^ T cells in immunotherapy-resistant melanoma and TNBC patients and that this complex is not present in melanoma patients capable of responding to immunotherapy. We also show that LSD1 has a direct effect on T cells by controlling EOMES nuclear entry and retention through the bivalent post-translational modification at lysine 641 to promote T cell exhaustion. This discovery has three important implications. First, that the EOMES^+^PD-1^+^ population needs no longer be regarded as an immunotherapy resistant population (12, 60, 61), as we can target the enriched nLSD1p axis in immunotherapy-resistant CD8^+^ T cells. Second, that any LSD1-targeting therapeutic intervention must disrupt the nuclear LSD1p axis or the LSD1-EOMES nuclear complex, and it appears that phenelzine or EPI-111 will be effective in this regard. Third, that T cell exhaustion and responsiveness to immunotherapy can be identified clinically through nuclear expression of LSD1 and our novel post-translational modifications of EOMES. Our findings that EOMES lysine 641 acetylation is especially suppressed in immunotherapy-responsive patients and significantly increased in immunotherapy-resistant patients raises the enticing prospect of a potential mechanism by which nuclear LSD1 may carry out its T cell dysfunctional program and serve as a target to overcome immunotherapy resistance. This, supports recent studies showing that LSD1 inhibition renders resistant melanoma cancer cells sensitive to anti-PD1 treatment *in vivo* (22). While LSD1 traditionally regulates gene expression via chromatin-associated PTMs, we have shown that nLSD1p regulates the nuclear dynamics of EOMES via demethylation or acetylation of target lysine residues, similar to its role in the regulation of nuclear dynamics of the non-histone protein p53 (13).

We propose that EOMES PTMs exert a bimodal function in T cells (Supplementary Figures 7A,B). In our model, the post-translational state of EOMES at lysine 641 or 373 regulated by nuclear LSD1p is critical in determining the functional or dysfunctional status of CD8^+^ T cells. Our model is supported by mutational analysis, LSD1p inhibition, and liquid biopsy profiling. The model proposes that dysfunctional and exhausted T cells as seen in immunotherapy-resistant patients are globally enriched in EOMES lysine 641 acetylation and nLSD1p with CoREST. Conversely, effector T cells have globally reduced LSD1p, no EOMES lysine 641 acetylation, and globally enriched expression of EOMES lysine 641/373 methylation. Naïve or immunotherapy-resistant patients with LSD1p:EOMES-Ac complexes maintain effector genes in a poised, repressive state; however, upon T cell activation, in responders, or on LSD1 inhibition, EOMES switches partners to become methylated and allow transcription factor recruitment for effector function.

In summary, we have identified for the first time that specifically targeting nLSD1p and the CoREST complex is superior to FAD-domain inhibitors such as GSK2879552 and that this is essential for reducing CSC signatures in solid tumors. Not only can exhausted CD8^+^ T cells be reinvigorated by treatment targeting the nuclear EOMES-LSD1 axis, but EOMES methylation and acetylation states can be used to discriminate between resistant and responsive disease.

Future clinical studies in larger cohorts of patients could exploit liquid biopsies to determine if EOMES is a true biomarker for treatment with LSD1 inhibitors and immunotherapy. It would also be useful to delineate how EOMES modulates these effects using ChIP sequencing to analyze chromatin binding interactions. Efforts are now required to define the precise mechanism by which the LSD1:EOMES complex orchestrates a dysfunctional signature, such as with genome-wide studies to establish the precise gene addresses of EOMES post-translational modifications in T cells as well as the optimal nuclear inhibitor or combinations of inhibitors.

## Data Availability Statement

All datasets generated for this study are included in the article/Supplementary Material.

## Ethics Statement

The studies involving human participants were reviewed and approved by ACT Health Research Ethics and Governance Office. The patients/participants provided their written informed consent to participate in this study. The animal study was reviewed and approved by The Australian National University Animal Experimentation Ethics Committee.

## Author Contributions

WT and RM wrote the manuscript together with SR. RM, WT, and SR designed the experiments. SR conceived the study. WT carried out carried out NanoString analysis, patient-related FACS analyses, and RNA-Seq experiments. RM carried out *in vitro* inhibitor experiments together with JDu and carried out the downstream imaging and tissue staining as well as designing and carrying out the digital pathology assays on patient samples. AT carried out the *in vivo* mouse experiments. KH carried out bioinformatics analyses, and NS provided advice on HIV data. SA, DY, LM, and TP provided clinical samples and patient stratification advice for the manuscript, and JDa and EB provided processed tissues and input into the manuscript. JF and ST provided LSD1-associated structural data and carried out binding assays. All authors contributed to the article and approved the submitted version.

## Conflict of Interest

In accordance with NHMRC policy and our ethical obligations as researchers, we report that, SR, RM, AT, WT, JDu, JDa, and JF have a financial interest in EpiAxis Therapeutics Pty Ltd. SR is also Chief Scientific Officer of EpiAxis Therapeutics Pty Ltd. We also have in place a plan for managing any potential conflicts arising from that involvement. The remaining authors declare that the research was conducted in the absence of any commercial or financial relationships that could be construed as a potential conflict of interest.
