# Supplementary material for: Targeting Nuclear LSD1 to Reprogram Cancer Cells and Reinvigorate Exhausted T Cells via a Novel LSD1-EOMES Switch
Source: Front Immunol. 2020 Jun 16;11:1228. doi: 10.3389/fimmu.2020.01228 (PMC7309504; doi:10.3389/fimmu.2020.01228)
Supplement: Supplementary file 1 [file Data_Sheet_1.docx]

Supplementary Materials

# Supplementary Methods

# Plasmids: The NLS region of LSD1 (UniProtKB O60341; aa104-129) with an N-terminal TEV cleavage site (ENLYFQS) was codon optimized for expression in *Escherichia coli* and synthesized (Genscript, Piscataway, NJ). This construct was inserted into a pGEX4T-1 vector, which contains a GST tag and ampicillin resistance, at the BamHI and EcoRI cloning sites. Mutagenesis was performed to generate the LSD1 NLS S111E and K114A mutants. Full length IMPα1 (UniProtKB P52292), IMPα3 (UniProtKB O00629), IMPα7 (UniProtKB O60684) and IMPβ(UniProtKB Q14974) were cloned into the PMCSG21 vector, which contains a 6xHis tag and spectinomycin resistance, at the SSPI site. LSD1 (UniProtKB O60341; aa104-852) was also cloned into the pMCSG21 vector at SSPI site and mutagenesis was performed to generate S111E and K114A mutants. IMPα3 lacking the auto-inhibitory IBB domain (UniProtKB O00629; aa64-521), and IMPα7 lacking the auto-inhibitory IBB domain (UniProtKB O60684; aa73-536) were codon optimized for *E. coli* expression, synthesized, and cloned into the pET15b vector at NdeI and EcoRI sites. The mouse IMPα1 lacking the auto-inhibitory IBB domain (UniProtKB P52293; aa71- 529) was cloned into pET30a vector at BamHI and EcoRI sites as described previously (62, 63).

# Recombinant Expression and Purification: Plasmids were transformed into BL21 (DE3) pLysS cells and expressed using the Studier auto-induction method (64). Starter cultures were inoculated into 2 L baffled flasks containing 500 mL of expression medium consisting of 1% w/v tryptone, 0.5% w/v yeast extract, 0.5% glycerol, 0.05% glucose, 0.2% w/v α-lactose, 0.025 M NH_4_SO_4_, 0.05 KH_2_PO_4_, 0.05M Na_2_HPO_4_, 1 mM MgCl_2_, and either 100 μg/mL ampicillin or 50 μg/mL kanamycin. Cells were incubated at 25°C and at 90 rpm overnight, then harvested via centrifugation at 6000 rpm and 18°C for 30 min and resuspended in phosphate buffer (PB; 20 mM imidazole, 300 mM NaCl, 50 mM phosphate pH 8.0) or tris-buffered saline (TBS; Tris pH 8.0, 125 mM NaCl) buffer with complete EDTA-free protease inhibitor (Roche). Cells were lysed using two freeze-thaw cycles and addition of 20 mg lysozyme and 0.5 mg DNase.

# Affinity purifications of 6xHis-tagged IMPα were performed by injecting clarified cell lysate onto a HisTrap 5 mL column using PB, washing the column with 15 column volumes and then eluting over 5 column volumes using a gradient elution with high imidazole (500 mM imidazole, 300 mM NaCl, 50 mM phosphate pH 8.0). Affinity purifications of GST-tagged proteins were performed on a GST Trap 5 mL column using TBS, and, after washing with TBS, 20 mg of IMPα was injected to allow interaction with LSD1 to occur. Further washing was performed with TBS to remove excess IMPα and the complex was eluted from the column using TBS supplemented with 10 mM glutathione. All size exclusion purifications were performed on a Superdex 200pg 26/600 column using TBS, and eluted proteins were pooled and concentrated using 10 kDa MW centrifuge filters to 10 mg/mL and stored at -80°C.

# Crystallization: All crystals were obtained using the hanging drop vapor diffusion method (3 μL drop size of 1:1 protein to reservoir mix) over a 300 μL reservoir solution. The IMP complexes with LSD1-NLSs (WT, S111E) were crystallized with 0.7 M sodium citrate, 0.01M DTT, 0.1M sodium HEPES pH 7, with rod-shaped crystals forming within 2-3 days. The IMPα1ΔIBB:LSD1-NLS complex crystallized in 0.7 M sodium citrate, 0.01 M DTT, 0.1M sodium HEPES pH 7 with a rod morphology that diffracted to 2.5 Å. The IMPα1ΔIBB:LSD1-NLS (WT, S111E) complexes both diffracted to 2.1 Å.

# Data collection and processing: X-ray diffraction data were collected at the Australian Synchrotron on the MX1 and MX2 macromolecular beam lines using an ASDC Quantum 210r and Quantum 315r detector, respectively (65, 66). Data reduction and integration were performed using iMosflm (67) for data collected using the ADSC210r detector. Merging, space group assignment, scaling, and truncation of 5% Rfree reflections were performed using Aimless (68, 69) and the CCP4 suite (70) Phasing was performed using molecular replacement in Phaser MR (71), with 5KLR (72) used as a search model for the IMPα1ΔIBB:LSD1-NLS complex. This model was then used to phase the IMPα1 LSD1 phosphorylation mutant complex S111E. The IMPα 3 LSD1 NLS complex was phased using 5XZX (73) as a search model. All models were refined using iterative cycles of coot (74) and phenix refine (75).

# Gel filtration assay: For each purification, a total of 1 mg protein was loaded and molar concentrations of 1:1 were used to assess interactions between IMPα and LSD1 proteins. The GE Superdex 200pg 16/600 column was pre-equilibrated in TBS at a run rate of 0.5 mL/min. For each purification, 1 mL fractions were collected and examined by SDS-PAGE to confirm the shift in LSD1 protein elution upon complex formation or to confirm no shift in elution for the absence of a formed complex.

# Microscale thermophoresis: A Monolith NT.115 instrument (NanoTemper Technologies) was used to perform microscale thermophoresis (MST). Purified IMPα1 in 20 mM HEPES, 125 mM NaCl, pH 8.0 was labeled using the NHS RED NanoTemper 2nd Generation labeling kit according to the manufacturer's instructions. For the affinity assay, 10 μL of labeled protein was mixed with 10 μL of L1 and cL1 at various concentrations. All samples were applied to Monolith NT Standard Treated Capillaries (NanoTemper Technologies), and thermophoresis was measured at 25°C with laser off/on/off times of 5 s/30 s/5 s. Experiments were conducted at 20% LED power and 40% MST infrared laser power. Data from independently performed experiments were fitted to the binding graph using the NT Analysis software version 1.5.41 (NanoTemper Technologies).

# Custom antibodies: Custom polyclonal rabbit EOMES-641k-Ac, EOMES-641k-Me2, and EOMES-373k-Me2 were generated by Mimotopes^TM^. Briefly, for antibody generation, a cysteine was incorporated at the C-terminus of the peptide (for peptide sequences please see Supplementary Table 3) and reacted to conjugate the peptide to an immunogenic carrier protein Keyhole Limpet Hemocyanin (KLH). No special immunization protocols were required to generate anti-methylated or anti-acetylated peptide antibodies. Rabbits for each peptide sequence were immunized several weeks apart. The first immunization was with an emulsion of the peptide conjugate with complete Freund’s adjuvant, the second using incomplete Freund’s adjuvant. Potent anti-peptide sera were obtained after several weeks. Methylated/acetylated-peptide antisera were conveniently tested using an enzyme-linked immunosorbent assay (ELISA), where the sera were titrated on microtiter plates coated with non-methylated-peptide and methylated-peptide or non-acetylated and acetylated peptide.

# For methylated/acetylated peptide antibody enhancement, the non-methylated/non-acetylated analogue of the peptide used for the immunization was coupled to a gel Sulfo Link (Thermo Fisher Scientific 20401:05273) using the available cysteine residue following the manufacturer’s instructions. The resultant gel was incubated with aliquots of the antisera to absorb antibodies specific to the non-methylated/non-acetylated peptide. The resultant antiserum had enhanced specificity for the methylated peptide or acetylated peptide sequence.

# To produce affinity purified antibodies specific to the methylated or acetylated peptide only, it was necessary to first perform enhancement to remove antibodies from the serum to the non-methylated/non-acetylated peptide. Specificity of the affinity purified antibodies was tested by ELISA back onto both the non-methylated and the methylated peptides/non-acetylated and acetylated peptides coated onto the plate. ELISA results are depicted in Supplementary Figure 8, showing specificity of the antibodies to their targets, and Supplementary Tables 5-7, showing the specificity and blank/negative control readouts for specific ELISA high dilutions (1/25000).

The results for the 1:25000 antibody dilution are shown in **Supplementary Tables 5-7**. These tables demonstrate that the ELISA blank and negative controls used for each antibody showed no binding to the associated antibody. Each PTM antibody was used in the ELISA to label its unmodified sequence (doubling as the antibody negative and specificity control) and to target the PTM modified form. The antibodies raised against the specific PTM forms were only able to detect the corresponding PTM form of the motif. At a representative high dilution (1/25000), AB-641-Ac only detected 641k-Ac, AB-641-Me2 only detected 641k-Me2, and neither were able to detect the unmodified 641k. The same was seen for AB-373k-Me2, which only detected 373k-me2 and not the unmodified target 373k. This approach used blanks and negative controls in the ELISA to show that the antibody signals were not background or non-specific and additionally used the unmodified motif as a negative control for the antibodies targeting 641k or 373k, which they did not detect, only the specific PTM they were raised against.

# Immunofluorescence staining: Immunofluorescence microscopy was performed to determine the mean TNFI, TCFI, and PCC as previously described (21). PCC values were determined by the strength of the relationship between two fluorochrome signals. Primary antibodies were: rabbit anti-human Ki67 (AB15580, Abcam, Cambridge, UK), mouse anti-human IFN-γ (AB212474, Abcam), goat anti-human TNF-α (DS-PB-02322, RayBiotech, Peachtree Corners, GA), rabbit anti-mouse LSD1 (05-939; Merck Millipore, Burlington, MA), rabbit anti-human EOMES (AB23345, Abcam), goat anti-human PD1 (AB36151, Abcam), mouse anti-human TBET (AB91109, Abcam), mouse anti-human CSV (H00007431-M08; Abnova, Taiwan), rabbit anti-human LSD1-s111p (ABE1462; Merck Millipore), goat anti-human SNAI1 (sc-10433; Santa Cruz Biotechnology, Dallas, TX), rabbit anti-human ALDH1A1 (ab52492; Abcam), goat anti-human ABCB5 (ab77549; Abcam), mouse anti-human CD133 (130-092-395; Miltenyi Biotech), mouse anti-human TIGIT (MAB7898; R&D Systems, Minneapolis, MN), rabbit anti-human LAG3 (AB227579; Abcam), goat anti-human TIM3 (AB47997; Abcam), rabbit anti-mouse LSD1 (05-939; Merck Millipore), rat-anti-rat/mouse CD8 (AB22378, Abcam), rat-anti rat/human CD8 (Ab60076, Abcam). Secondary antibodies used were anti-rabbit-Alexa Fluor 488 (A21206; Life Technologies, Carlsbad, CA) or anti-rabbit-Alexa Fluor 568 (A10042; Life Technologies), anti-mouse-Alexa Fluor 568 (A10042; Life Technologies), anti-goat-Alexa Fluor 633 (A21082; Life Technologies), or anti-rat-Alexa Fluor 647 (A-21472; Life Technologies).

# Supplementary Figures


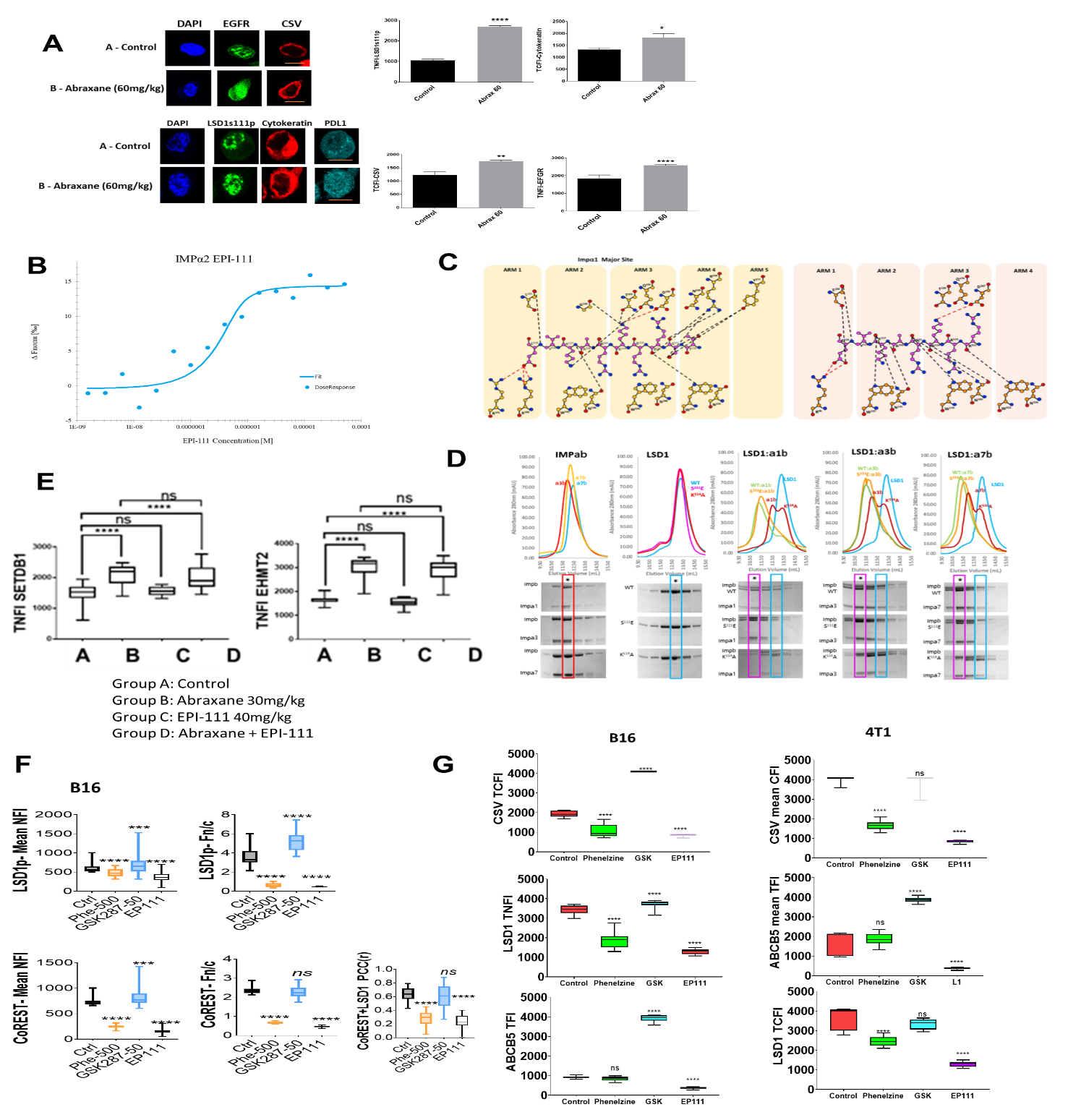


**Supplementary Figure 1.** (A) Cancer cells isolated from the MDA-MB-231 xenograft model were treated with either control or Abraxane and IF microscopy performed probing with primary antibodies targeting CSV, EGFR, LSD1s111p, cytokeratin, and PD-L1 with DAPI. Representative images for each dataset are shown. Graphs plots represent the mean TNFI/TCFI measured using ImageJ minus background (n≥20 cells per group). Scale bar is shown in orange and is equal to 10 μm in length. (B) Binding curve of IMPα2 L1(blue) using the signal from Thermophoresis + T-jump microscale thermophoresis experiments. (C) Structure of LSD1 NLS IMPα1 (left) and LSD1 NLS IMPα3 (right). Interaction schematic, hydrogen bonds shown in black dashed lines, salt bridge interactions shown in red dashed lines. Binding residues are shown in stick representation, IMPα residues orange, LSD1 residues magenta. (D) Gel filtration and SDS-PAGE analysis of LSD1 IMPα/β complexes. Gel filtration of three IMPα/β complexes, α1/β (yellow), α3/β (red), α7/β (blue), and LSD1 aa104-852 WT (blue), phosphorylation mutant S111E (magenta), and methylation mutant K114A (red). Each IMPα/β complex was tested against the three LSD1 constructs for complex formation, LSD1 WT apo (blue), WT (green), S111E (orange), K114A (maroon). Asterisk shows the gel filtration peak on SDS-PAGE. IMPα/β peak highlighted on SDS-PAGE gel with red box, LSD1 peak highlighted with blue box, LSD1:IMPα/β complexes highlighted with magenta box. (E) MDA-MB-231 cells were treated with control scrambled peptide, Abraxane, EPI-111, or combination Abraxane+EPI-111, fixed, and labelled with antibodies for SETDB1 and EHMT2. TNFI was measured for each. Graphs represent the TNFI and Fn/c for n = 20 or more cells (n = 3 experiments). Mean NFI and Fn/c are shown. Mann-Whitney test, ****p<0.0001, ***p=0.0002, **p=0.0021, ns >0.05. (F) B16 cells were treated with phenelzine, GSK, EP-111, or control scrambled peptide, fixed, and labeled with primary antibodies targeting LSD1p and CoREST, and the TNFI values were measured using ImageJ (n>20 cells/group). Graphs plot the TNFI of LSD1 and CoREST, Pearson’s correlation coefficients (PCC) between LSD1 and CoREST, and the Fn/c (ratio of nuclear to cytoplasmic staining: below 1 is cytoplasmic biased, above 1 is nuclear biased). IF analysis. Mann-Whitney test, ****p<0.0001, ***p=0.0002, **p=0.0021, *p=0.033, ns >0.05. (G) 4T1 and B16 cells were treated either with vehicle control, 500 μM phenelzine, EPI-111, or 50 μM GSK287 and screened by IF microscopy with primary antibodies targeting CSV, LSD1p, and ABCB5 with DAPI. Graph represents the TNFI values for LSD1p, EGFR and TCFI for CSV, PDL1, and cytokeratin calculated using ImageJ-Fiji (n>20 individual cells).


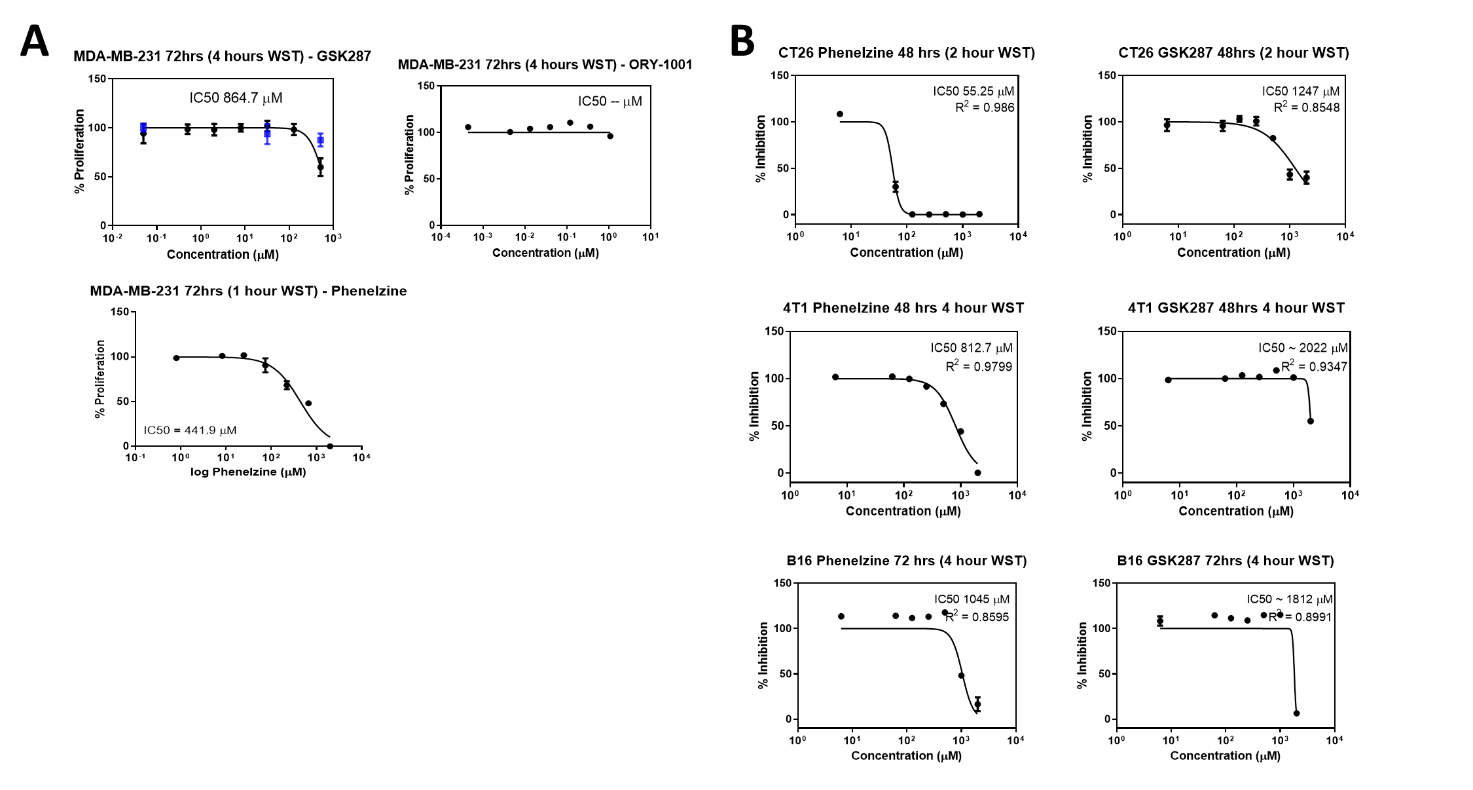


**Supplementary Figure 2.** (A) MDA-MB-231 cells were treated with control, GSK287, ORY-1001, or phenelzine as assessed using the WST-1 proliferation assay with the % effect on proliferation plotted. (B) Cancer cell lines CT26 or 4T1 were treated with control, GSK287, or phenelzine as assessed using the WST-1 proliferation assay with the % effect on proliferation plotted.


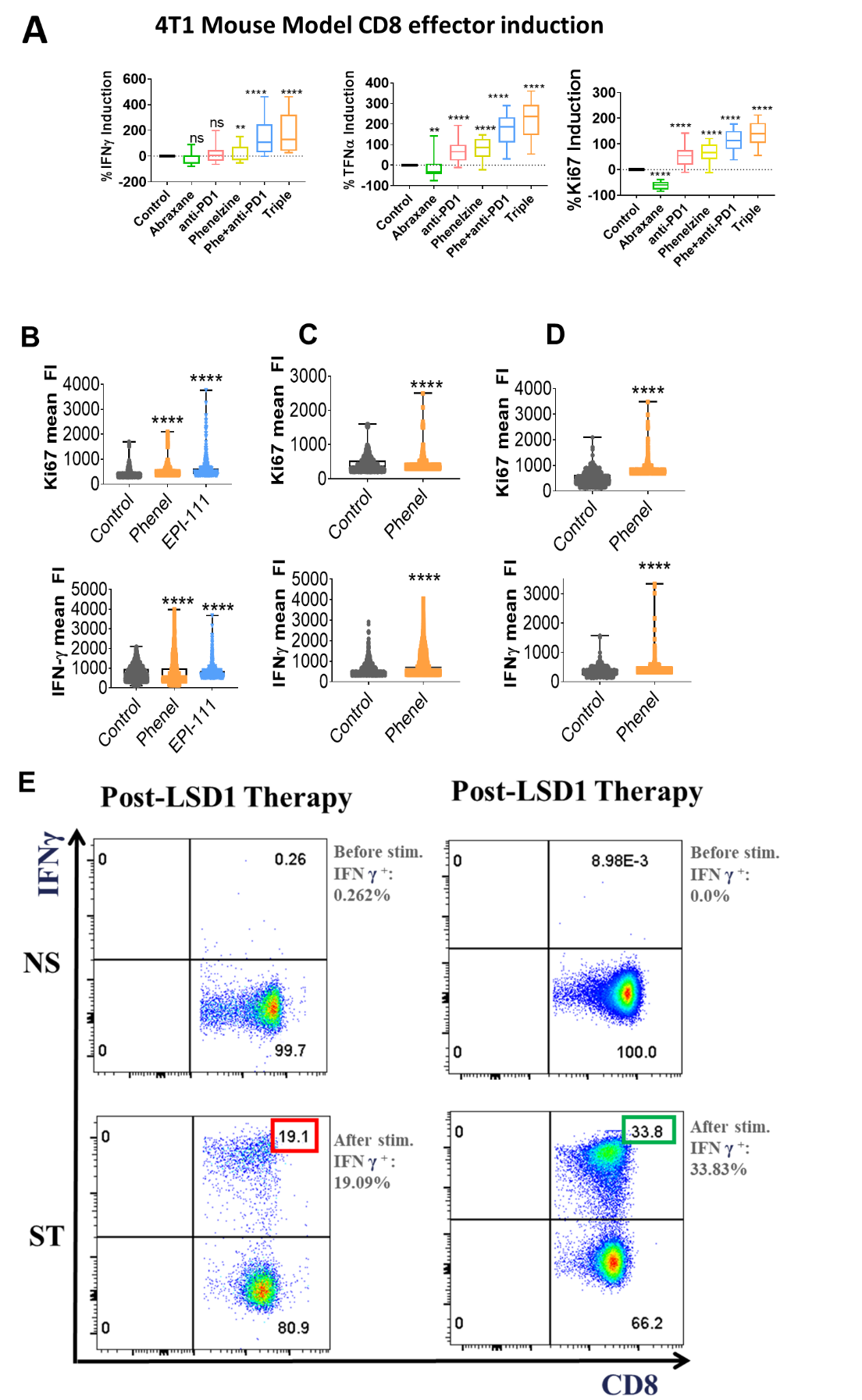


**Supplementary Figure 3.** **A**) 4T1 Mouse TNBC model-derived tumour samples were cytospun and IF microscopy performed probing with primary antibodies to CD8, IFN-γ, TNF-α, and Ki67 with DAPI. Graphs plots represent the mean % change of each marker measured using ImageJ minus background (n≥20 cells per group). **B**) CD8^+^ T cells isolated from a TNBC patient were untreated or treated with phenelzine or EPI-111 prior to stimulation. Samples were subjected to digital pathology with primary antibodies targeting Ki67 or IFN-γ with DAPI (n>100 individual cells). Graphs plot the mean fluorescent intensity for each marker with statistical differences calculated with the Mann-Whitney test, ****p<0.0001, ns>0.05**. C**) CD8^+^ T cells isolated from an ER/PR^+^/HER2^-^ patient were untreated or treated with phenelzine prior to stimulation. Samples were subjected to digital pathology with primary antibodies targeting Ki67 or IFN-γ with DAPI (n>100 individual cells). Graphs plot the mean fluorescent intensity for each marker with statistical differences calculated with the Mann-Whitney test, ****p<0.0001, ns>0.05**. D**) CD8^+^ T cells isolated from a PD melanoma patient were untreated or treated with phenelzine prior to stimulation. Samples were subjected to digital pathology with primary antibodies targeting Ki67 or IFN-γ with DAPI (n>100 individual cells). Graphs plot the mean fluorescent intensity for each marker with statistical differences calculated with the Mann-Whitney test, ****p<0.0001, ns>0.05**.** **E**) CD8^+^ T cells were isolated from a TNBC patient either pre or post LSD1 therapy. FACS analysis of IFN-γ expression was performed on patient-derived TNBC CD8^+^ T cells before and after PMA/ionomycin stimulation.


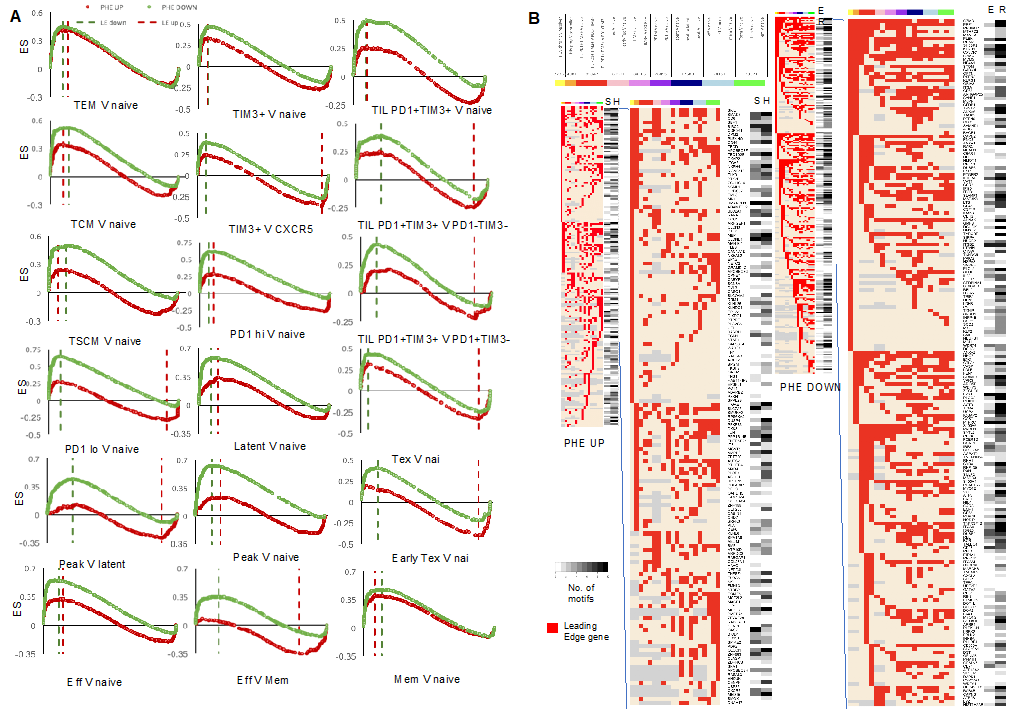


**Supplementary Figure 4.** (A) Enrichment plots of the phenelzine up- and down- regulated gene signatures. Plots show individual genes (dots) from the two phenelzine regulated signatures distributed across the ranking in expression from x to y (x versus (V) y). LE: leading edge cut-off. ES: enrichment score. Tex: Exhausted T cell. TIL: tumor-infiltrating lymphocyte. Eff: effector T cell. Mem: memory T cell. Tscm: stem cell memory T cell. Tcm: central memory T cell. Tem: effector memory T cell. (B) Genes in the phenelzine up- and down-regulated signatures that are leading edge genes in each study as per Figure 4B. The number of SMAD (S) and HIC2 (H) or EOMES (E) and RUNX1 (R) motifs in nearby enhancers (from (Philip et al., 2017)) are marked. Red: leading edge gene. Grey (not measured in study). GSE: Gene Expression Omnibus series number. Tex: exhausted T cell. TIL: tumor-infiltrating lymphocyte. Eff: effector T cell. Mem: memory T cell. Tscm: stem cell memory T cell. Tcm: central memory T cell. Tem: effector memory T cells. Note genes are considered LE in the direction of enrichment.

**
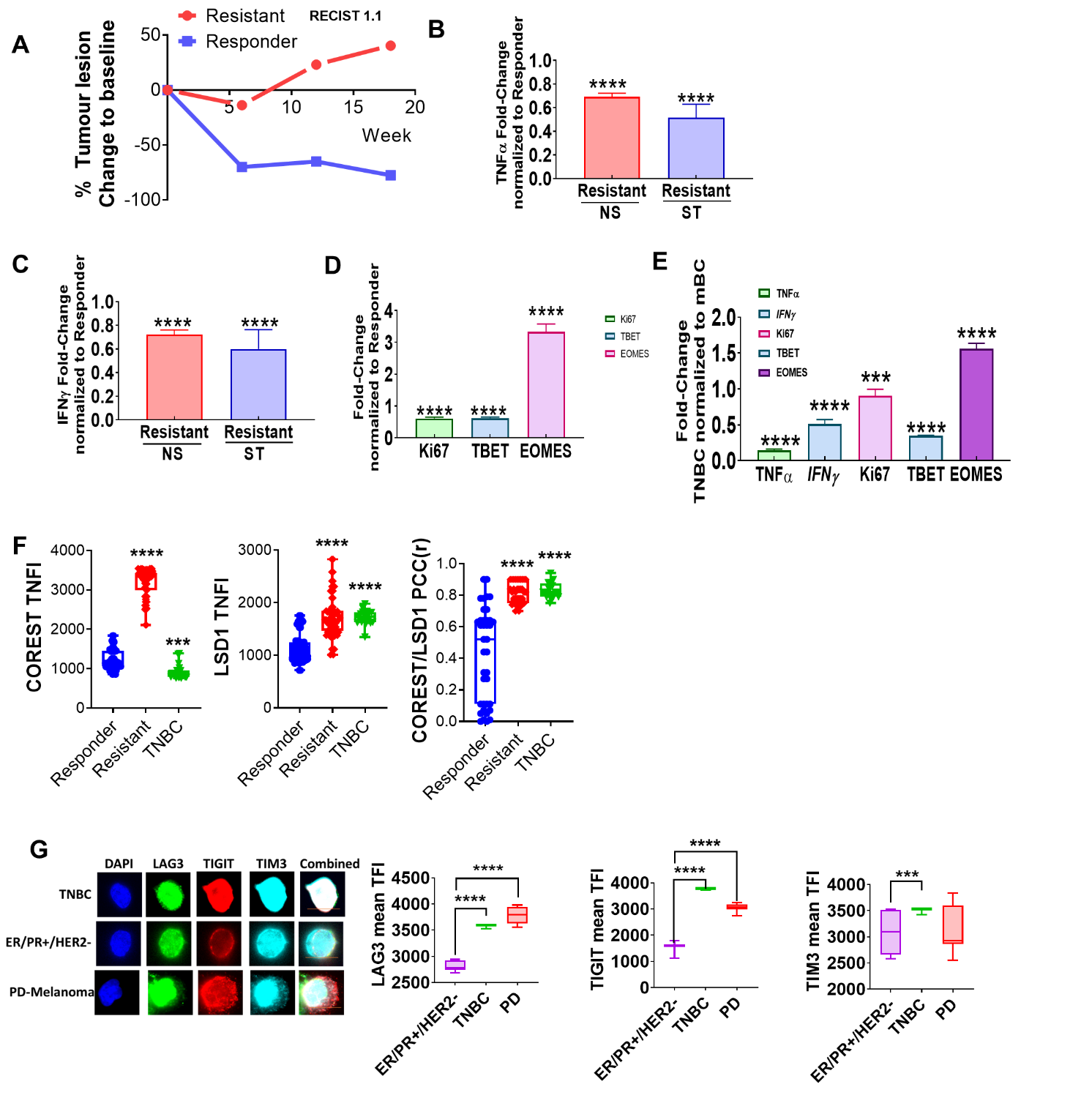
**

**Supplementary Figure 5.** (A) Melanoma patients were classified based on objective response to immunotherapy (either mono or dual therapy with pembrolizumab, nivolumab, and/or ipilimumab) into complete responders (CR) or progressive disease (PD). The depicted plot shows the % change in tumor growth as described for RECIST 1.1 for either PD (progressive disease) or CR (complete responders) cohorts. (B) to (D) TNF-α, IFN-γ, and Ki67 or TBET and EOMES were labelled on responder and resistant derived melanoma CD8^+^ T cells. Total fluorescence intensity (TFI), total cytoplasmic fluorescence (TCFI) and total nuclear fluorescence intensity (TNFI) values were measured using ImageJ (n>20 cells/patient, n=10 patients/cohort) and used to calculate the % change relative to responder patient cohorts. Graphs represent the % change. (E) TNF-α, IFN-γ, and Ki67 or TBET and EOMES were labelled on CD8^+^ T cells derived from ER^+^/PR^+^/HER2^-^ or TNBC breast cancers. Total fluorescence intensity (TFI), total cytoplasmic fluorescence (TCFI), and total nuclear fluorescence intensity (TNFI) values were measured using ImageJ (n>20 cells/patient, n=10 patients/cohort) and used to calculate the % change relative to responder patient cohorts. Graphs represent the % change. (F) CD8 T cells were isolated from TNBC or melanoma patients (responder and resistant) and subjected to IF microscopy with primary antibodies targeting CoREST and LSD1. Graph represents the mean NFI for CoREST and LSD1 measured in ImageJ. PCC between LSD1 and CoREST in melanoma patients with PD (resistant) or CR (responsive) or TNBC was calculated with ImageJ and plotted based on >20 cells/patient (n=10 patients/cohort; 4 samples/patient). Mann-Whitney test, ****p<0.0001, ***p=0.0002, **p=0.0021, *p=0.033, ns > 0.05. (G) CD8 T cells were isolated from PD melanoma patients, ER/PR‑/HER2^‑^, and TNBC patients and subjected to immunofluorescence microscopy with primary antibodies targeting TIGIT, LAG3, and TIM3. Graphs represent the mean TFI values for LAG3 and TIM3 and the mean CFI values for TIGIT measured using ImageJ (n>20 cells per a patient, n=10 patients per cohort). Representative images for each group are shown.

**
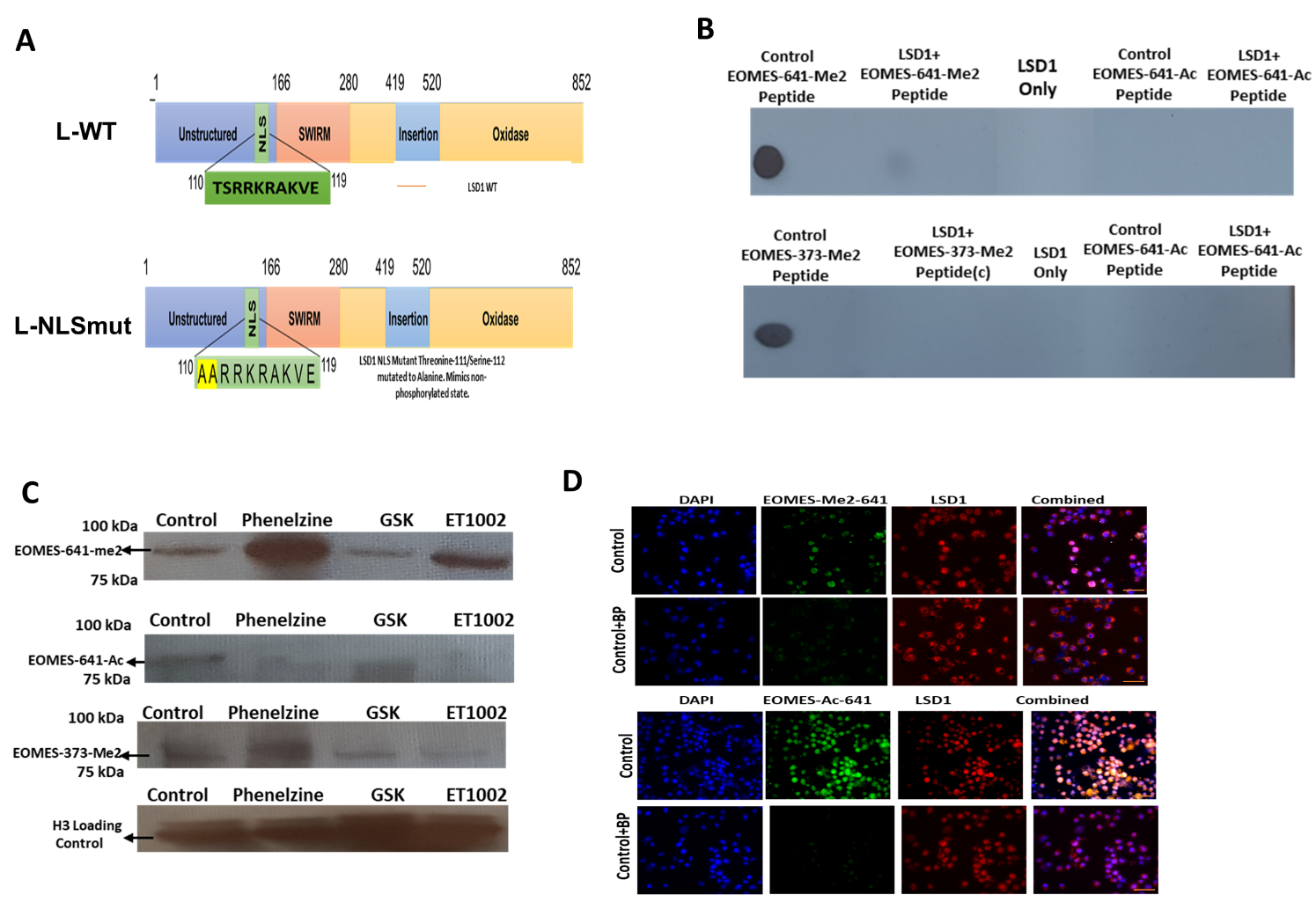
**

**Supplementary Figure 6.** (A) Schematic of the LSD1 WT and NLS mutant plasmids. (B) Peptide pools specific for specific EOMES PTMs of either Lysine-641-Me2, Lysine-641-Ac, or Lysine-373-Me2 were treated with a recombinant LSD1 enzyme and subjected to dot blot analysis with specific antibodies targeting EOMES-641-Me2 or EOMES-373-Me2. Images depict example dot blots for n=3 dot blots. (C) Immunoblot analysis of nuclear extracts of Jurkat T cells treated with control, phenelzine, EPI-111, or GSK were carried out. Immunoblots were probed with EOMES-641-Me2, EOMES-641-Ac, EOMES-373-Me2, or H3 loading control. Blots are a representative image of n=3 immunoblots. (D) IF microscopy was performed on Jurkat T cells fixed and either treated with control or a peptide specific for the corresponding antibody target and probed with antibodies targeting LSD1 and EOMES-641k-Me2 or EOMES-641k-Ac with DAPI. Example wide-field images are shown demonstrating loss of staining in peptide blocking samples.


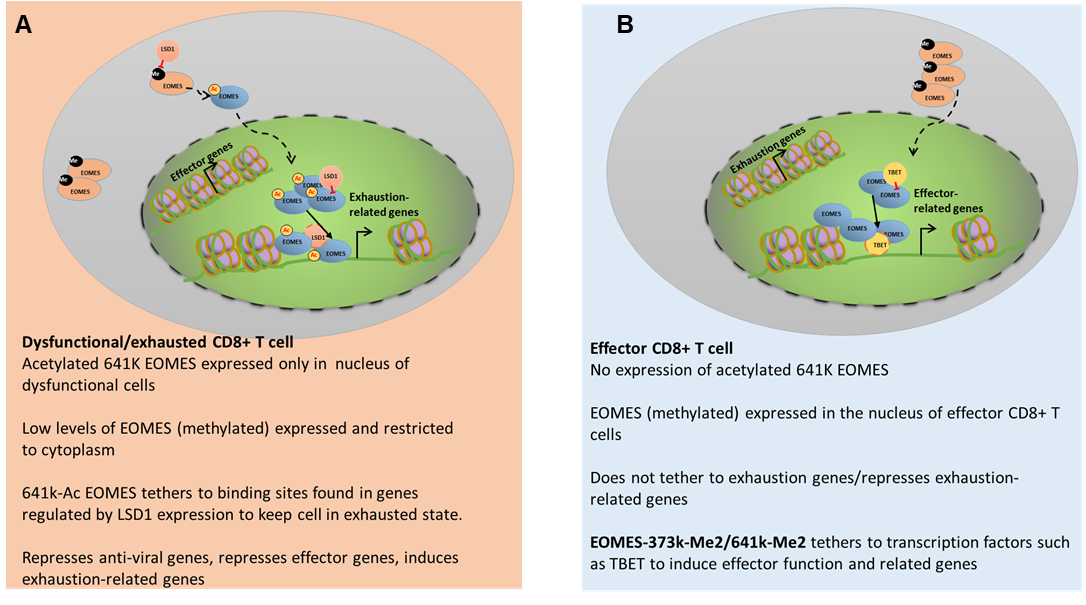


**Supplementary Figure 7.** Model of EOMES PTM mediated CD8+ T cell exhaustion. A) is the model for the dysfunctional T cell signature, regulated 641-EOMES-Ac and B) is the model for the effector/functional T cell regulated by 641-EOMES-Me2.

A

B

C

D

E

**Supplementary Figure 8.** Five ELISA graphs showing the specificity of the custom antibodies designed for the EOMES 641 lysine motif with either no PTM (A) or with Me2/Ac 641K (B), Me2 at 614K (C), 373 lysine motif with no PTM (D), or the EOMES DNA-binding motif with (E) Me2 at 373K. Rabbits 1 and 2 were used to raise antibodies against the unmodified peptides, Rabbits 3 and 4 were used to raise antibodies against the Lysine-641-Me2 antibody, Rabbits 5 and 6 were used to raise antibodies against the Lysine-641-Ac antibody, Rabbits 7 and 8 were used to raise antibodies against the Lysine-373-unmodified peptides, and Rabbits 9 and 10 were used to raise antibodies against the Lysine-373-Me2 peptides. The ELISA data clearly indicate that the antibodies raised against the PTM forms of EOMES are specific only for the specific PTM and do not recognize or bind to the unmodified peptide (which also works as a negative control for the specificity of the antibodies).

# Supplementary Tables

**Supplementary Table 1.** The enriched gene ontology and pathways in the phenelzine up- and down- regulated signature gene lists.


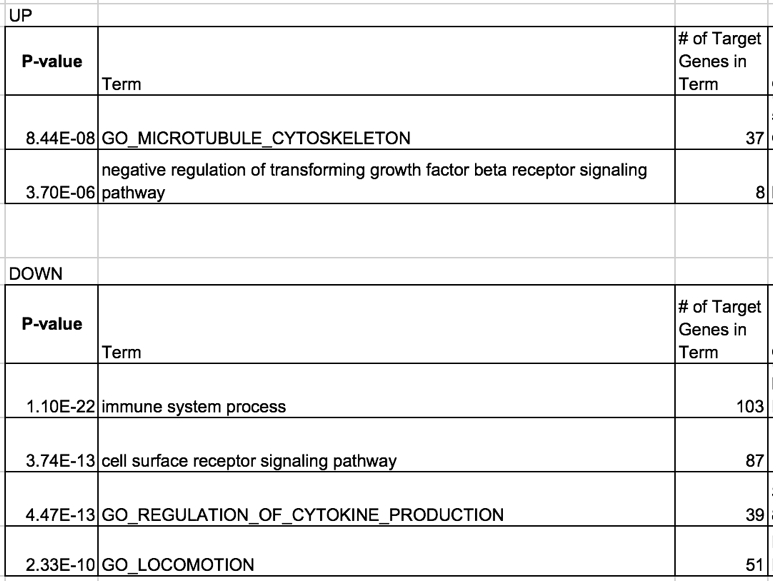


**Supplementary Table 2.** Liquid biopsy sampling: number of samples, sampling regimen, and treatment. Top: melanoma patients; bottom, breast cancer patients.

| **Patient Cohort** | **Patient Samples** | **Sampling regimen per patient** | **Treatment (Rx)** |
| --- | --- | --- | --- |
| **Healthy Donor (HD)** | 10 Donors | 1 Sample taken every two weeks (total 4 samples) | **-** |
| **Responder (By RECIST v 1.1)** | 25 Patients | 1 Sample taken every two weeks (total 4 samples) | Ipi, Nivo or Pembro |
| **Resistant (By RECSIT v 1.1)** | 15 Patients | 1 Sample taken every two weeks (total 4 samples) | Ipi, Nivo or Pembro as first line and after resistance Nivo and Ipi combination therapy followed |

| **Patient Cohort** | **Patient Samples** | **Sampling regimen per patient** |
| --- | --- | --- |
| **Stage IV Metastatic ER+/PR+/HER2-** | 15 Patients | 1 Sample taken every two weeks (total 4 samples) |
| **Stage IV metastatic TNBC** | 15 Patients | 1 Sample taken every two weeks (total 4 samples) |

**Supplementary Table 3.** Novel antibody motif peptides.

**Supplementary Table 4.** Mouse and Humans EOMES NLS motifs.

| **Supplementary Table 5. ELISA results for custom antibody targeting PDL1-641-Lysine-Me2 at 1:25000 dilution.** | | | | | |
| --- | --- | --- | --- | --- | --- |
| Antibody | 1:25000 AB Dilution | Blank | Negative | Unmodified Peptide | Modified Peptide 641-K-Me2 |
| Unmodified 641k | Rabbit 1 | 0.053 | 0.058 | 1.032 |  |
| Unmodified 641k | Rabbit 2 | 0.058 | 0.061 | 0.678 |  |
| 641k-Me2 | Rabbit 3 | 0.055 | 0.065 | 0.091 | 1.103 |
| 641k-Me2 | Rabbit 4 | 0.058 | 0.053 | 0.121 | 1.036 |
| **Supplementary Table 6. ELISA results for custom antibody targeting PDL1-641-Lysine-Ac at 1:25000 dilution.** | | | | | |
| Antibody | 1:25000 AB Dilution | Blank | Negative | Unmodified Peptide | Modified Peptide 641-K-Me2 |
| Unmodified 641k | Rabbit 1 | 0.062 | 0.068 | 1.036 |  |
| Unmodified 641k | Rabbit 2 | 0.058 | 0.059 | 1.135 |  |
| 641k-Ac | Rabbit 5 | 0.056 | 0.067 | 0.071 | 0.704 |
| 641k-Ac | Rabbit 6 | 0.053 | 0.066 | 0.065 | 0.677 |
| **Supplementary Table 7. ELISA results for custom antibody targeting PDL1-373-Lysine-Me2 at 1:25000 dilution.** | | | | | |
| Antibody | 1:25000 AB Dilution | Blank | Negative | Unmodified Peptide | Modified Peptide 373-K-Me2 |
| Unmodified 373k | Rabbit 7 | 0.058 | 0.057 | 0.836 |  |
| Unmodified 373k | Rabbit 8 | 0.057 | 0.068 | 0.824 |  |
| 373k-Me2 | Rabbit 9 | 0.053 | 0.063 | 0.125 | 0.652 |
| 373k-Me2 | Rabbit 10 | 0.054 | 0.055 | 0.111 | 0.622 |
